# Evaluation of a large-scale weight management program using the consolidated framework for implementation research (CFIR)

**DOI:** 10.1186/1748-5908-8-51

**Published:** 2013-05-10

**Authors:** Laura J Damschroder, Julie C Lowery

**Affiliations:** 1 HSR&D Center for Clinical Management Research, VA Ann Arbor Healthcare System (152), 2215 Fuller Rd, Ann Arbor, MI 48105, USA

**Keywords:** Implementation, Qualitative methods, Obesity, Conceptual framework

## Abstract

**Background:**

In the United States, as in many other parts of the world, the prevalence of overweight/obesity is at epidemic proportions in the adult population and even higher among Veterans. To address the high prevalence of overweight/obesity among Veterans, the MOVE!® weight management program was disseminated nationally to Veteran Affairs (VA) medical centers. The objective of this paper is two-fold: to describe factors that explain the wide variation in implementation of MOVE!; and to illustrate, step-by-step, how to apply a theory-based framework using qualitative data.

**Methods:**

Five VA facilities were selected to maximize variation in implementation effectiveness and geographic location. Twenty-four key stakeholders were interviewed about their experiences in implementing MOVE!. The Consolidated Framework for Implementation Research (CFIR) was used to guide collection and analysis of qualitative data. Constructs that most strongly influence implementation effectiveness were identified through a cross-case comparison of ratings.

**Results:**

Of the 31 CFIR constructs assessed, ten constructs strongly distinguished between facilities with low versus high program implementation effectiveness. The majority (six) were related to the inner setting: networks and communications; tension for change; relative priority; goals and feedback; learning climate; and leadership engagement. One construct each, from intervention characteristics (relative advantage) and outer setting (patient needs and resources), plus two from process (executing and reflecting) also strongly distinguished between high and low implementation. Two additional constructs weakly distinguished, 16 were mixed, three constructs had insufficient data to assess, and one was not applicable. Detailed descriptions of how each distinguishing construct manifested in study facilities and a table of recommendations is provided.

**Conclusions:**

This paper presents an approach for using the CFIR to code and rate qualitative data in a way that will facilitate comparisons across studies. An online Wiki resource (http://www.wiki.cfirwiki.net) is available, in addition to the information presented here, that contains much of the published information about the CFIR and its constructs and sub-constructs. We hope that the described approach and open access to the CFIR will generate wide use and encourage dialogue and continued refinement of both the framework and approaches for applying it.

## Background

In the United States, as in many other parts of the world [1], the prevalence of overweight/obesity is at epidemic proportions in the adult population [2] and even higher among Veterans [3]. Nearly three-fourths of the 5.7 million Veterans [4] who receive their medical care from the Veterans Health Administration (VHA) are overweight or obese [3]. Overweight and obesity are associated with substantial morbidity and mortality [5-8] and increased healthcare costs for patients, healthcare systems, and payers [7,9,10]. In 2001, VHA primary care providers cited effective weight management programs as the most pressing need in preventive services for Veterans [11].

Veteran Affairs (VA) National Center for Health Promotion and Disease Prevention (NCP) designed MOVE! as a patient-centered, multi-tiered set of tools and treatment options based on published guidelines for obesity management [11-14]. A comprehensive set of implementation guides was developed by NCP for local facilities (http://www.move.va.gov).

Dissemination of the MOVE! weight management program in a network of 155 medical centers and 872 community-based outpatient clinics made this the largest and most comprehensive dissemination of a weight management program in the U.S. [11,15]. In the first year of the program, only about 8 per 1000 Veterans who were candidates for MOVE! (body mass index more than 30 kg/m^2^, or between 25 to 30 kg/m^2 ^with one or more obesity-related chronic health conditions, *e.g.*, hyperlipidemia [11]) actually participated in the program. In the second year of the program, local facilities varied widely in the number of candidate Veterans who participated in MOVE!, from no participants at many facilities to a high of 37 participants per 1,000 MOVE! candidates [15]. MOVE! cannot help Veterans if it is not implemented as designed.

The present study was conducted 18 to 22 months after initial dissemination of MOVE!, with the first aim of applying the Consolidated Framework for Implementation Research (CFIR) [16] to identify contextual influences that explain the wide variation in implementation success experienced by VA medical facilities. Though the program has progressed significantly since that time, our findings are nonetheless helpful for other large-scale program disseminations. Our second aim is to illustrate, step-by-step, how to apply the CFIR to identify influential contextual constructs on implementation and to suggest refinements to the framework, related methods, and directions for future research.

## Conceptual framework

Our approach to evaluating implementation of the MOVE! program in VA is rooted in realist philosophy [17]. The basic tenet of this approach indicates that the MOVE! program will alter context within and surrounding each medical center (*e.g.*, trigger formation of a new interdisciplinary team), which will trigger mechanisms that will result in intended and unintended outcomes. We seek to unpack and understand the complex and dynamic influences at play. Use of theory-based and pre-specified constructs will help to generalize findings and make them more easy to integrate with findings from other studies to build a stronger evidence base to: identify factors that influence or predict implementation success; guide how to adapt programs and tailor implementation strategies; and provide a foundation for developing higher-order models and theories related to implementation [18]. Our approach to use of theory in this study can be described as theory-building, rather than applying a pre-constructed theory or model wholesale. We rely on a ‘menu of constructs’ approach which enables systematic and comprehensive exploration and identification of potential explanatory themes or variables to shed light on the complex social phenomenon of implementation [19]. Taking a menu-of-constructs approach also allows us to flexibly include only constructs that apply to the study at hand, which in turn allows us to limit the duration of our interviews to a reasonable time period.

The CFIR provides a comprehensive taxonomy of operationally defined constructs from multiple disciplinary domains (*e.g.*, psychology, sociology, organizational change) that are likely to influence implementation of complex programs [16]. CFIR constructs are organized into five major domains and, as applied to this study, are: characteristics of the MOVE! program (*e.g.*, evidence strength and quality, complexity); the outer setting (*e.g.*, patient needs and resources); inner setting (*e.g.*, compatibility of MOVE! with existing programs, leadership engagement); and the process used to implement the program (*e.g.*, quality and extent of planning, engagement of key stakeholders). The fifth domain, characteristics of individuals involved (*e.g.*, knowledge and attitudes), was not applied in this study because our focus was not on individual-level behavior change.

## Methods

Our evaluation of MOVE! implementation was done retrospectively; qualitative data were collected through semi-structured interviews of key local stakeholders over the telephone. The CFIR was used to guide development of the interview guide and data coding and analysis.

## Facility selection

We selected sites based on a two-step process. First, administrative data indicating the level of participation of candidate Veterans in the MOVE! program at each VA medical center in FY2007 was used to identify sites in the highest and lowest quartiles of participation. We purposively selected three sites within the highest quartile and two from the lowest quartile in a way that maximized variation by geographic location. Next, information about program components actually implemented and participation rates in the following year were used to characterize implementation effectiveness as high, low, or in transition. Two of the study facilities were characterized as having high implementation effectiveness. Both facilities were in the top quartile for both FYs and had robust MOVE! programs [20]. Two facilities were characterized as low implementation facilities. Though one of these facilities was in the top quartile in FY2007, it turned out ‘participation’ was limited to initial assessment. Veterans were then referred to a community-based program with no follow-up. Our fifth study site had almost no participants in FY2007, reflecting a failed implementation attempt in this first year; however, by the time of our interviews, they were in the midst of renewed implementation activities. The burgeoning success of their second attempt is reflected by increased participation reported in FY2008. A list of facilities and their key characteristics is provided in Table 1.

**Table 1:** Facility characteristics

**Implementation effectiveness**	**Active MOVE! treatment components**	**Number of MOVE! participants per 1,000 candidate veterans**	
		**FY2007**	**FY2008**
**Low**	**•** Initial assessment only	26.7	12.3
**Low**	**•** Initial assessment	3.8	3.4
	**•** Ten-week series of weekly group classes		
	**•** ‘Reunion’ group at end of each series		
**Transition**	**•** Initial assessment	0.4	7.1
	**•** Piloted six-week series of weekly group classes		
	**•** Ad hoc maintenance support		
**High**	**•** Initial assessment; limited self-management support	19.2	19.4
	**•** Ten-week series of weekly group classes		
	**•** Ad hoc post-completion support		
**High**	**•** Initial assessment; limited self-management support	27.6	37.7
	**•** Six-week series of weekly group classes		
	**•** Therapy using pharmacological agents		
	**•** Intensive outpatient lifestyle counseling program		
	**•** Bariatric surgery		

## Study participants

Interviews were conducted between July and October 2007. Thirty-two potential staff were invited to participate in the study; 75% (n = 24) agreed to participate. The regional and local facility MOVE! coordinators were identified from a centrally available list and were interviewed first. We used a snowball sampling technique by asking the local facility coordinators to identify staff who were involved with delivering or implementing MOVE! at their facility [21]. A waiver of signed informed consent was granted by the VA Ann Arbor Healthcare System Institutional Review Board (2007-050289) in compliance with Helsinki Declaration standards. Participants were verbally consented at the start of the telephone interview; permission to record the interview was also requested. Participants were offered a $10 gift card as a token of appreciation for their time.

## Data collection procedures

The qualitative portion of the semi-structured interview guide is provided in Additional file 1. After a short series of closed-ended questions (described elsewhere; [15]), we asked open-ended questions, eliciting descriptions of each respondent’s role and how MOVE! was implemented at their facility. We probed aspects of their narratives to understand how each CFIR construct manifested at their facility. Our research question was focused on understanding context. We focused our questions and analysis on collective perceptions of the program, inner and outer setting, and aspects of the process of implementation. Thus, we did not analyze the data for individual characteristics that are related to individual behavior change. We encouraged open narrations to elicit information the interviewee deemed important and to minimize recall bias [22]. The principal investigator (LJD) led all the interviews. At least one other team member also participated to help ensure all topics were covered and responses were fully understood. All interviews were digitally recorded and transcribed verbatim.

## Qualitative data coding and case memos

**Figure 1:**
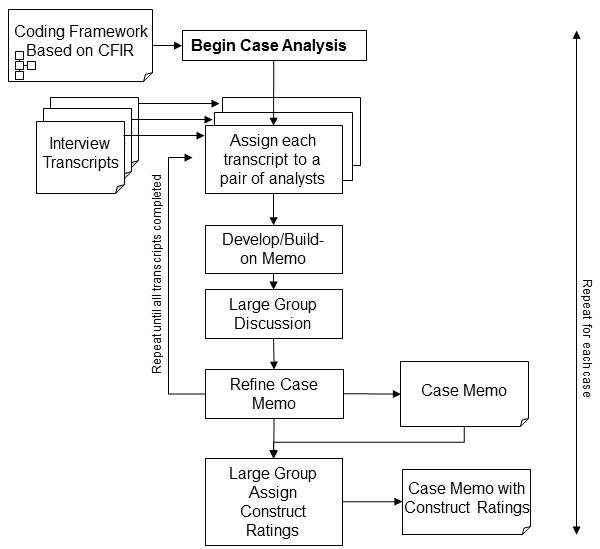
Team-based work flow for case analyses.

Figure 1 shows a schematic of the coding and analysis process. We used a content analysis [23] and largely deductive approach, using the CFIR as a coding framework [16]. Additional guidance for coding each construct is available online (http://www.wiki.cfirwiki.net). We were also open to new themes that may have arisen inductively from the data. Our coding process was guided by consensual qualitative research methods [24,25]. The consensual research approach has the following features: data were collected through open-ended questions in semi-structured interviews; multiple judges were used throughout data analysis to foster multiple perspectives; consensual validation was achieved through a process of deliberation and consensus [26]; and an outside auditor (a qualitative expert not integrally involved in the study) reviewed the process to help maximize validity of findings. More detail about this approach is described elsewhere [15].

A summary memo was developed for each facility (case) using a two-level deliberated consensus approach. First, a pair of analysts independently coded an individual transcript. They then met together and compared their coding, discussed differences, and agreed on final codes. Based on these codes, they wrote a case memo, organized by CFIR construct; each construct had summary statements with supporting quotes (an example memo with excerpts and a template are available online (http://wiki.cfirwiki.net/index.php?title=General:CFIR_in_action). Pairs of analysts continued to code all of the transcripts for a single case using this process, adding to and refining the memo until all transcripts were coded and the case memo was complete. During this process, each new transcript was used to confirm previously written summary statements, document counterpoints, or add new information for each construct. The entire study team (two pairs of analysts, the two authors, and, periodically, our qualitative expert) met weekly to review the case memos as they evolved. Each pair of analysts presented their contributions to the memo and the larger team reviewed, deliberated, and modified the memo as appropriate. Thus, each case memo was developed through an evolving two-level consensus process. This process resulted in five memos, one for each study facility.

## Rating the CFIR constructs

The five case memos were each subjected to a rating process. The large group team used a deliberated consensus process to assign a rating to each construct within each facility. Table 2 lists the criteria used to guide assignments of the ratings. The ratings reflect the valence (positive or negative influence) and the magnitude or strength of each construct in each facility based on the case memos. When all constructs for all cases were rated (using a case-oriented approach, because ratings were applied within each case), we compared ratings for each construct across cases (using a variable-oriented approach, because each construct is compared across cases) to help ensure consistent application of ratings. This approach combines the strengths of a case-oriented method, which allows for rich context-specific consideration when rating each construct, with a variable-oriented method, which promotes identifying patterns and relationships by construct across cases to heighten overall validity of ratings [21].

**Table 2:** Criteria used to assign ratings to constructs

**Rating**	**Criteria**
−2	The construct is a negative influence in the organization, an impeding influence in work processes, and/or an impeding influence in implementation efforts. The majority of interviewees (at least two) describe explicit examples of how the key or all aspects (or the absence) of a construct manifests itself in a negative way.
−1	The construct is a negative influence in the organization, an impeding influence in work processes, and/or an impeding influence in implementation efforts. Interviewees make general statements about the construct manifesting in a negative way but without concrete examples:
	• The construct is mentioned only in passing or at a high level without examples or evidence of actual, concrete descriptions of how that construct manifests;
	• There is a mixed effect of different aspects of the construct but with a general overall negative effect;
	• There is sufficient information to make an indirect inference about the generally negative influence; and/or
	• Judged as weakly negative by the absence of the construct.
0	A construct has neutral influence if:
	• It appears to have neutral effect (purely descriptive) or is only mentioned generically without valence;
	• There is no evidence of positive or negative influence;
	• Credible or reliable interviewees contradict each other
	• There are positive and negative influences at different levels in the organization that balance each other out; and/or different aspects of the construct have positive influence while others have negative influence and overall, the effect is neutral.
+1	The construct is a positive influence in the organization, a facilitating influence in work processes, and/or a facilitating influence in implementation efforts. Interviewees make general statements about the construct manifesting in a positive way but without concrete examples:
	• The construct is mentioned only in passing or at a high level without examples or evidence of actual, concrete descriptions of how that construct manifests;
	• There is a mixed effect of different aspects of the construct but with a general overall positive effect; and/or
	• There is sufficient information to make an indirect inference about the generally positive influence.
+2	The construct is a positive influence in the organization, a facilitating influence in work processes, and/or a facilitating influence in implementation efforts. The majority of interviewees (at least two) describe explicit examples of how the key or all aspects of a construct manifests itself in a positive way.
	Missing Interviewee(s) were not asked about the presence or influence of the construct; or if asked about a construct, their responses did not correspond to the intended construct and were instead coded to another construct. Interviewee(s) lack of knowledge about a construct does not necessarily indicate missing data and may instead indicate the absence of the construct.

## Analysis and interpretation

A matrix was created that listed the ratings for each CFIR construct for each of the facilities. We focused this part of our analysis on discerning patterns across the two high and the two low implementation facilities. We aligned the two facilities with low implementation effectiveness together to compare and contrast them to the two facilities with high implementation effectiveness. This allowed us to identify patterns in ratings of the CFIR constructs that distinguished between high and low implementation effectiveness—*i.e.*, that were qualitatively correlated with implementation effectiveness. Constructs were characterized as: missing too much data to discern a pattern, not distinguishing between low and high implementation facilities, or weakly or strongly distinguishing low versus high implementation facilities.

The following excerpt of a case memo highlights a single construct (Relative Advantage) showing the construct rating (+2), a summary statement, and a series of supporting statements and quotes:

Relative Advantage (+2)

Summary Statement: Stakeholders saw the benefits of MOVE! as a means to extend weight loss programming beyond the preexisting weight management class. Moreover, stakeholders knew bariatric surgery was going to be implemented in the VISN and MOVE! helped achieve the pre-surgery behavioral requirements for surgical candidates. Perception of benefits is also demonstrated by early (early-adopter) implementation status of facility.

MOVE! Coordinator: Specifically, she saw the benefits of MOVE! over their previous weight management program and felt that it helps prepare candidates for their new bariatric surgery program:

• *Well prior to spring of ’05, we’ve had tentative MOVE! Level 1, it’s kind of like a weight management 101 type class. We just turned our old weight management class into the MOVE! Level 1 class.*

• *…unfortunately, well back then it was more like we had nothing else to offer and so they’d attend the class and when you could tell they were, you know, newly motivated or wanted additional information, at that point, we had nothing more to offer.*

• *So I think what we liked about the organization of the whole program on a national level is historically where our struggle has been is as a dietician, we aren’t really supposed to be heavily into exercise physiology and psychology. I mean you can touch on some of that stuff but historically when we’ve tried to get something a little more multi-disciplinary in terms of true weight loss management, it’s been hard. So the MOVE! program to me was exciting. [So before the MOVE! program came in, were you able to find a physical therapist or occupational or recreational therapist to work with? Or was it the MOVE! program that facilitated that connection?] The MOVE! program helped us there*

• *we knew we would have to offer a component of the Level 5, the bariatric surgery [I: Right] program, [VISN Office City] mandated that they attend MOVE! Level 1 and MOVE! Level 2 classes to get a lot of education, you know, pre-surgery so we knew that we would be having veterans because I already had veterans asking about the surgery so that was another good reason to put it in place but I would, my guess would be that I have less than, less than 5% of all my veterans in the MOVE! Level 2 classes are working towards surgery*

Dietitian Supervisor: The biggest strength of MOVE! is its inter-disciplinary emphasis.

• *…the biggest thing is the inter-disciplinary strength because the dieticians can not affect the need to change by themselves and even though we’ve been teaching weight control for years and years, the enhanced education for the patients by having those other people up there to support the mental health aspects of this and the exercise aspect as well as our end of it, to me is really the number one.*

The case memos were imported into NVivo Version 8 and then coded so that reports could be generated that contained all summary statements and supporting quotes for all the cases. The code reports provided rich detail to help understand how each construct manifested in the low and high implementation facilities. Findings were developed and used to craft recommendations based on practices at the high implementation facilities that appeared to contribute to positive ratings, while suggesting ways to mitigate or work-around constructs with negative influence (*i.e.*, barriers) at the low implementation facilities. Data from the transition facility were used to further confirm or contrast these findings qualitatively.

## Results

Of the 31 CFIR constructs assessed, 10 constructs strongly distinguished between facilities with low versus high MOVE! implementation effectiveness (See Table 3). Another two constructs exhibited a weak pattern in distinguishing low versus high implementation effectiveness. Sixteen constructs were mixed across facilities. The remaining two constructs had insufficient data to assess. The following sections briefly describe each construct, highlighting how they manifested in the study facilities. We also provide brief suggestions, insights, and reflections for coding constructs. Additional file 2 provides more detailed descriptions of the manifestation of those constructs strongly differentiated between low and high implementation facilities. Our online wiki provides more comprehensive coding and rating guidelines (http://wiki.cfirwiki.net/index.php?title=CFIR_Taxonomy).

**Table 3:** Ratings assigned to CFIR construct by case

	**Low implementation facilities**		**Transition facility**	**High implementation facilities**		
**Site ID**						
**I. INTERVENTION CHARACTERISTICS**	**200**	**500**	**100**	**300**	**400**	
Intervention Source^a^	E	E	E	E	I	
**Evidence Strength and Quality**	**-2**	+1	+1	**+2**	+1	
**Relative Advantage**	**-2**	+1	+1	**+2**	**+2**	**
**Adaptability**	**-2**	**+2**	+2	**+2**	**+2**	
**Trialability**	0	0	+1	+1	0	
**Complexity** (reverse rated)	Missing	-2	-2	-2	**+2**	
**Design Quality and Packaging**	-2	+2	+1	+1	+1	
**Cost**	0	0	0	0	0	
**II. OUTER SETTING**						
Patient Needs and Resources	**-2**	0 (mixed)	+1	**+2**	**+2**	**
Cosmopolitanism	0	0	+1	0	0	
Peer Pressure	0	0	0	0	0	
External Policy and Incentives	-1	**-2**	N/A	0	+1	*
**III. INNER SETTING**						
**Structural Characteristics**	Missing	Missing	Missing	Missing	Missing	
**Networks and Communications**	**-2**	**-2**	+1	**+2**	**+2**	**
**Culture**	Missing	Missing	Missing	Missing	Missing	
**Implementation Climate**						
Tension for change	0	0	+2	+1	+1	**
Compatibility	-2	+1	0	+1	+2	
Relative priority	-1	**-2**	-2	+1	**+2**	**
Organizational Incentives and Rewards	+0	-1	0	0	+1	
Goals and Feedback	**-2**	-1	+1	+1	**+2**	**
Learning climate	Missing	-1	Missing	+1	**+2**	**
**Readiness for Implementation**						
Leadership Engagement	**-2**	-1	+2	**+2**	**+2**	**
Available resources	**-2**	**-2**	-1	+1	-1	*
Access to knowledge and information	-1	Missing	Missing	**+1**	-1	
**V. PROCESS**						
**Planning**	-1	Missing	+1	+1	+1	**
**Engaging**						
Opinion Leaders	Missing	Missing	Missing	Missing	Missing	
Formally Appointed Internal Implementation Leaders	-1	**+2**	+2	**+2**	**+2**	
Overall Championing	-1	**+2**	+2	+1	**+2**	
Physician Champion	**-2**	**+2**	0	+1	+2	
Other Champions			+2	+2		
External Change Agents	0	0	0	0	0	
**Executing**	N/A	N/A	N/A	N/A	N/A	
**Reflecting and Evaluating**	-1	**-2**	+1	+1	**+2**	**

** Construct strongly distinguishes between low and high implementation effectiveness.

* Construct weakly distinguishes between low and high implementation effectiveness.

^a^ I: Treated MOVE! as internally developed E: Treated MOVE! as externally developed.

## Intervention characteristics domain

### Intervention source

This was not a distinguishing construct because it did not vary across sites; most of the study facilities regarded MOVE! as an externally developed program. The one exception was a high implementation facility that embraced MOVE! as a welcome extension to their already-existing weight management program. Note that although the program was actually developed externally, it is important to code based on local stakeholders’ perceptions of the program’s source. Thus, the site that viewed MOVE! as an extension of their existing program perceived the source as more internal than external. We coded this construct neutrally as ‘internal’ or ‘external’ rather than assigning a positive or negative numeric rating, because we were interested in whether the perceived source varied across sites, not in whether that source was viewed positively or negatively.

### Evidence strength and quality

This was not a distinguishing construct. Though one of the low implementation facilities had a particularly strong negative view of the evidence supporting MOVE! (‘we had a difficult time selling chief of staff and chief of medicine on the efficacy of the pilot study’) and one of the high implementation facilities had a strongly positive perception of the same evidence, the other three facilities had a weakly positive perception of the evidence that was vaguely expressed. Some interviewees cited evidence from the literature, some inferred MOVE! should be successful because they thought other weight loss programs were (*e.g.*, Weight Watchers), and some believed camaraderie arising from group visits would be helpful for their Veterans. It is important to note that evidence may come from multiple sources, not just published scientific literature.

### Relative advantage

Relative advantage was a strongly distinguishing construct. The two high implementation facilities both had strongly positive perceptions about the advantages of MOVE!. Even the facility with the most well-developed weight management program welcomed MOVE! because of the additional visibility and attention it brought to their weight management program:

‘With the help of MOVE! information, MOVE literature, MOVE! Whatever […] it sort of boosted [our existing program] more and we were able to expand more.’ [MOVE!Coord; 400]

The transition site also appreciated the interdisciplinary nature of MOVE!:

‘We didn’t have all the components that we needed [for weight management] […] before MOVE! came along, we were in a silo so we did our part as a dietician so we were just working out of our corner of the world.’ [MOVE!Coord; 100]

One of the low implementation facilities thought that a community-based wellness program was better than MOVE! and opted to refer patients there rather than implement MOVE!:

‘The community program has] these walking classes, these swim classes, these things are in the community […] [and it relies] more heavily on personal responsibility of the participants.’ [MOVE!Coord; 200]

### Adaptability

This was not a distinguishing construct because it manifested as a strong positive influence in four of the five facilities. We heard about many examples of how MOVE! was adapted to their facility; *e.g.*, variation in the number of sessions, duration of the classes, exercise approaches, patient screening process, class content, and whether groups were ‘closed’ (no new patients were allowed to join until the start of a new series of classes) or ‘open’ (patients could join anytime), and where the program was administered—*e.g.*, within primary care or nutrition and food services.

### Trialability

This was not a distinguishing construct. Most sites did not conduct a trial; but because our interviews took place well after implementation had started, it was difficult for interviewees to recall whether this was because the program didn’t allow it (-1 rating) or that it was allowed, but the site decided it wasn’t necessary (0 rating). Only one site actually did a trial (+1 rating) and a second study site mentioned learning from the experience of another site in their region who participated in the national pilot study of MOVE! (+1 rating).

### Complexity

This was not a distinguishing construct because all sites viewed the implementation of MOVE! as a complex process. MOVE! requires staff participation from multiple service lines/professions (*e.g.*, dietitians, health psychologists) to set up substantial, new infrastructure to support group visits, new processes to screen and refer patients, etc. The single facility that regarded MOVE! as relatively simple had the most well-developed pre-existing weight management program.

It is important to note that we ‘reverse rated’ this construct to be consistent with the other constructs; *i.e.*, a positive sign denotes a perception of a less complex implementation and a negative sign indicates a more complex implementation.

### Design quality and packaging

This was not a distinguishing construct because four of the five facilities were consistent in finding materials and support from the national coordinating office to be helpful.

One of the low implementation facilities was especially appreciative of supporting materials:

‘We pulled from all of the MOVE! literature which there’s just a magnitude of information […] I think they did an excellent job in preparing the handouts, preparing the literature, preparing the calendars, the banners. The banners are so nice. We have one hanging in the end of our hallway; we have one in our room, the classroom. The videos are good, you know, I think that’s excellent.’ [MOVE Coord; 500]

We included perceptions of supporting materials and guides within this construct. Other programs will be ‘packaged’ differently; so, operationalization of this construct will vary widely, depending on the type of program or intervention.

### Cost

This was not a distinguishing construct because cost was not an explicit consideration. The more salient construct was available resources (see below), because of constraints in available staff time and classroom space.

## Outer setting domain

### Patient needs and resources

This construct strongly distinguished between low and high implementation facilities. Staff members at the high implementation facilities were quite knowledgeable and passionate talking about their Veterans. The MOVE! teams showed patients they cared in many ways and were responsive in designing the program to meet patient needs. One high implementation site talked about bringing in specialists who addressed topics based on patient needs:

‘When the patients see a doctor come in and we have a diabetes specialist that comes in once every six weeks and we have a person who does a presentation on sleep apnea. When they see these people, they get more enthusiastic about what they need to do […] I think that’s a motivator just seeing that all these healthcare providers, especially doctors […] just present something, they’re just eager to come every week.’ [Dietitian; 400]

One patient was so enthused about the program that he recruited other Veterans to come to MOVE! and had a list of ‘between 50 and 100 patients’ who agreed to try MOVE!.

Staff at both of the low implementation facilities mentioned that a barrier for patients was the co-pay (an out-of-pocket payment for the service) required to attend MOVE! classes (this co-pay has since been waived). Staff at one low implementation facility thought that patients would be concerned about losing VA healthcare benefits if they got healthier by losing weight.

However, at this same low implementation facility, the coordinator and physician champion did actively seek and respond to patient suggestions; for example, to provide longer-term support:

‘We have what’s called the reunion week and we have Veterans who are coming back who started the program over a year ago. They’re able to come in, with the effort I think to motivate those who are in the current group by telling them how they’ve been successful at changing their habits, how they’ve been successful at maintaining their weight loss, how they’ve had improvements in overall health, decreased medications.’ [Physician Champion; 500]

This facility was rated as 0 (neutral) overall. Some neutral ratings reflect consistently neutral influences, but for this case we assigned a neutral rating because of mixed positive and negative influences, which balanced each other.

To code and rate this construct, we looked for evidence of staff not just demonstrating knowledge of their patients’ needs and resources but also the degree to which this knowledge was acted upon to better align the MOVE! program with existing processes.

### Cosmopolitanism

This was not a distinguishing construct. Most people we talked to had few or no contacts outside their facility. The exception to this was the transition site where the MOVE! coordinator was recently hired from another facility and retained professional ties with several other coordinators. She was able to draw on this network to learn about and apply strategies that worked elsewhere (rating of +1).

### Peer pressure

Peer pressure was neutral at all of the facilities because none of the interviewees had anything to offer on this topic. These study sites, being publicly supported medical centers, were not subject to competitive or market pressures. At this early stage of MOVE! implementation, there was also an absence of urgency or pressure to implement to keep up with other VA sites.

Though interviewees were not asked directly about peer pressure, open-ended questions gave ample opportunity for influences of peer pressure to be expressed, and it was clear that these influences were absent (*i.e.*, neutral influence). This should be contrasted with a code of ‘missing’ in the event that we do not have any data to determine the nature of the influence.

### External policy and incentives

This construct weakly distinguished between high and low implementation facilities. External policies and incentives is a relatively broad construct. In the context of this study, VHA performance measures were the source of prevailing influence within this construct. The performance measures mentioned most often were those associated with achieving targets related to physiologic measures; *e.g.*, getting blood pressure (BP) under control (*e.g.*, BP ≤130/80) in a targeted percentage of enrolled Veterans. At the time of this study, there were no performance measures directly related to MOVE!. Thus, the MOVE! coordinator at one high implementation facility wanted to link participation in MOVE! to changes in BP, blood cholesterol, and blood glucose, earning that site a +1 rating:

‘I requested […] that we would like to have another nurse assigned to our program […] to be able to […] record information about blood pressures and cholesterols and perhaps blood sugars […] I would like to show […] that weight loss has something to do with improving all of this.’ [MOVE!Coord; 400]

Performance measures related to BP and lipid control seemed to work at cross-purposes to MOVE! at the two low implementation facilities. For example, at one of the sites (-2 rating):

‘When executive management is being held accountable for certain parameters at the VA […] there is a performance measure about the lipid profiles and so […] that’s what drove the boat […] the big thing, the big conflict […] would be space […] we have hypertension group classes, we have hyperlipidemia group classes, we have pain management group classes and then there’s MOVE!.’ [MOVE!Coord; 500]

Another stakeholder at the same site highlighted the challenges of not having a performance measure related to MOVE! in a follow-up email:

’Even more important than [funding] is accountability. Our facility should be ranked/compared/rewarded/admonished based on its relative performance in this program. Anything measured will likely get its due attention/resources, and will likely improve.’ [Physician Champion; 500]

## Inner setting domain

### Structural characteristics

This construct comprises many of the traditional quantitative measures of context, including age and size of the organization. There was no mention of these aspects influencing implementation of MOVE!, perhaps because these measures are often proxies of more proximal factors. This construct also includes potential influences of the social architecture (*e.g.*, how people are organized into separate service lines or clinics to deliver health care). We found that the quality and nature of networks and communications within and across organizational units were the more proximal influence, as described in the next section.

### Networks and communication

This construct strongly distinguished between low and high implementation facilities. We identified three sub-themes that clearly distinguished low from high implementation facilities. First, the high quality of working relationships across service (*e.g.*, nutrition, primary care) and professional (*e.g.*, health psychologist, dietitian) boundaries was apparent in the high implementation facilities:

‘If I need something, I just contact either the primary care supervisor or the mental health RN supervisor and request a meeting and they’ve been cooperative.’ [MOVE!Coord; 300]

In the other high implementation site, the coordinator (a Nurse Practitioner in primary care) had strong working relationships with other primary care providers, which helped in coordinating care for a patient who was working hard to lose weight and stay off of medication:

‘[The patient] looks dejected [today], extremely depressed and […] he goes, ‘I’m trying so hard to lose weight and I just saw my primary doctor and my primary doctor put me on diabetic medication and I’m like trying to do all of this and I still have to start taking medication?’ so I said, ‘You know what? Let me go and take a look at your chart […] and see what […] is actually needed’ so I go and review the chart, I call this patient’s primary care provider and I said, ‘He’s under my supervision and I’m trying to get him to lose weight. Do you agree that we can give him three-month trial to fix this with diet?’ […] So he agreed!’ [MOVE! Coord; 400]

In contrast, at one low implementation facility, a former MOVE! coordinator was not even told that a new coordinator had been hired to replace her.

The second sub-theme was related to team formation. The MOVE! teams at the two high implementation and transition facilities met regularly, for example:

‘Every two weeks we meet after the MOVE sessions […] with all the members of our group to discuss successes and other things […] we do this through our […] supposedly lunch time […] we carve out like 20 minutes to 30 minutes maximum […] to discuss obstacles, to discuss problems.’ [MOVE!Coord; 400]

These regular meetings helped the team coalesce. Multiple members of the interdisciplinary teams confirmed the collaborative nature of their team:

‘From what I’ve seen out in the world […] there’s a huge friendliness attitude here […] We have a huge staff retention […] so we all know each other and have worked together […], So that’s a huge benefit for us […] nobody was real pushy or bossy or anything. We all kind of collaboratively worked together so that definitely helped.’ [Physical Therapist; 300]

In the low implementation facilities, communications were poor between staff involved with MOVE! and they did much of their communication through email, if at all:

‘[If we have any MOVE! team meetings] we haven’t been invited. I don’t think we do, though.’ [Librarian; 200]

The third sub-theme was related to communications about MOVE! to other staff and patients. Multi-pronged and on-going communications helped to ensure primary care providers continued to refer patients to MOVE! in the high implementation facilities:

‘Every now and then we’ll send them a blanket message to all providers reminding them about the MOVE! program and how they need to make the referrals to the MOVE! program. I’ve met several times with the LPNs at their monthly meetings, encouraging them as front line people that they need to sell the program and […] a lot of them have attended the class to see what it’s like.’ [MOVE!Coord; 300]

In the low implementation facilities, some patients presented themselves to MOVE! staff, confused about what MOVE! was; a movie, a dance class, or bariatric surgery were some of the assumptions:

‘Sometimes they come without that little hard copy consult and they think that they need to see a movie […] they’re kind of confused sometimes about what they’re coming for […] [Librarian; 200]

Thus, the two high implementation sites were rated a strong positive, and the two low implementation sites were rated a strong negative.

### Culture

We did not ask explicitly about perceptions of overarching culture.

### Implementation climate

This construct comprises six sub-constructs. Conceptually, the aggregate of these six constructs may provide an overall measure of implementation climate, but we rated the individual sub-constructs, which are more useful for generating actionable recommendations.

#### Tension for change

This construct strongly distinguished between high and low implementation facilities. There was no expressed need for the program—or expressed concern that the program was not needed—in either of the low implementation facilities (neutral rating). Staff at high implementation facilities expressed some dissatisfaction with their current weight management options and welcomed MOVE! as a way to fill some of the gaps in their programming:

‘We had nothing else to offer and so they’d attend the class and when you could tell they were newly motivated or wanted additional information, at that point, we had nothing more to offer.’ [MOVE!Coord; 300]

Tension for change was strongest at the transition facility after expectations were raised in the first year but the program failed to materialize:

‘For a year, it was […] stagnant […] they had put up the […] [MOVE!] posters, […] and they didn’t have anything set up so people were consulting to the MOVE program when there wasn’t even a program set up.’ [MOVE!Coord; 100]

#### Compatibility

This was not a distinguishing construct. Despite one low implementation site having a strong negative rating and one high implementation facility having a strong positive rating, two of the facilities (one high and one low implementation) had a weak positive rating based on general statements like, ‘[…] everybody believes in the program.’

Compatibility has two major themes: compatibility with stakeholder values and compatibility with existing processes. Related to the first sub-theme, one low implementation facility felt that programming for a community-based program was better aligned with their desire to provide a wider array of options that focused on wellness, versus MOVE!, which at that time was marketed to “obese Veterans.” Related to the second theme, MOVE! was perceived as being highly compatible in one of the high implementation sites with a pre-existing metabolic clinic which focused on weight management.

#### Relative priority

This construct strongly distinguished between high and low implementation facilities. At one high implementation facility, the high priority of getting a bariatric surgery program in place worked against MOVE! implementation efforts at first. However, the MOVE! coordinator successfully linked the success of the bariatric surgery program to success of MOVE!, which increased priority for MOVE!

In contrast, it was clear that the low implementation facilities were struggling to respond to other higher priority initiatives, such as setting up new traumatic brain injury and poly-trauma screening programs and an urgent push to reduce clinic backlogs:

‘We had such a backlog […] It just depends on where you are on the totem pole […].We are absolutely, pardon the expression, under the gun to take care of these returning Iraq Veterans and so it’s a matter of, the MOVE program’s important, but these people are on fire over here.’ [MOVE!Coord; 500]

#### Organizational incentives and rewards

This was not a distinguishing construct. There was little or no evidence of any monetary rewards or of less tangible incentives like positive evaluations at any of the study facilities. The absence of incentive was rated as a weak negative influence at one low implementation facility because an interviewee acknowledged that she could not expect to get a raise, a bonus, or a pat on the back for successfully implementing MOVE!. All other sites were rated as neutral.

#### Goals and feedback

This construct strongly distinguished between high and low facilities. All of the facilities struggled with collecting program data and translating it into useable information. Organizational leaders generally did not ask for program data. One supervisor in a low implementation facility tried to collect tracking data but was burdened by the lack of tools:

‘I haven’t pushed it [compiling data] because our clinical responsibilities are so high and pulling it together, we’ve worked on it every spare minute for the past two days […] just compiling the data on a hard copy.’ [MOVE!Coord; 500]

In contrast, at the high implementation facilities, coordinators regularly tracked program data and reported it to organizational leaders who reviewed progress of the program:

‘I know how they’re doing during my MOVE! level two classes because I keep track of their weight from week to week […]. We do every quarter look at all the surveys and my clerk kind of comes up with a report of all the questions, comments, outcomes and I send that to [the Physician Champion] quarterly.’ [MOVE!Coord; 300]

In addition, coordinators in both of the regions associated with the high implementation facilities used program data to keep the program visible with key regional-level leaders. One regional coordinator used program data to win funding for additional dedicated staff at all of the medical centers in the region, and the other regional coordinator coached local coordinators about how to use program data to argue for needed resources.

#### Learning climate

This construct strongly distinguished between high and low facilities. We did not assess all dimensions of learning climate, but an important theme that clearly arose out of the data was the difference in the degree to which interviewees felt psychologically safe to take initiative in implementing MOVE!. Both of the high implementation facilities exhibited multiple dimensions of a learning climate: MOVE! coordinators were not afraid to experiment; they shared ideas with peers and superiors; and they had regular forums for learning from others. For example, one regional coordinator rotated meetings between sites in the regions so people could get to know and learn from one another. Facility coordinators kept in touch with one another through email and phone as well:

‘[These connections] give me an idea of what they’re doing and how we can modify here and I can give them a few suggestions that we have […] we kind of share, exchange information and it really benefits both sides because people do things differently and we learn from each other.’ [MOVE!Coord; 400]

There were indications of a potentially toxic climate at one low implementation facility:

‘I contacted the next likely person […] he just seemed to be so enthused about our goals […] [so I] Focused him in my binoculars […] I sent him an email. I didn’t want any arrows in my back so […] the safest thing to do here is in the little email (laughing) and then if that gets positive response, then you actually meet someone.’ [MOVE!Coord; 500]

### Readiness for implementation

This construct comprises three sub-constructs. Conceptually, the aggregate of the three constructs may provide an overall measure of readiness for implementation; but like our approach for implementation climate, we rated the individual sub-constructs instead, which are more useful for generating actionable recommendations.

#### Leadership engagement

This construct strongly distinguished between high and low implementation facilities. Service chiefs at the high implementation facilities allocated time for their respective staff to be a part of the interdisciplinary team and the coordinators had supervisors who were actively supporting the program.

Leaders helped to solve problems, get the resources needed, and ensure MOVE! was visible in the organization:

’If we have any equipment issues or you know, space issues, although space, you know, is hard but you know, they continue to look for us and help and it’s kept up there on […] radar, so they haven’t forgot about it […] if you say, ‘Can you bring it up at this meeting’ and that, they certainly will […] I would say leadership here is supportive and interested in it and then by them agreeing to hire, to hire a two positions for this MOVE program I think says a lot. It’s saying yes we will support you, we have value into the program.’ [Supervisor; 300]

Leaders at one low implementation facility seemed to work against implementing MOVE!, in part because MOVE! was low priority and in part because they were so focused on developing a bariatric surgery program and failed to acknowledge MOVE!’s role in preparing candidate patients for the surgery. At the other low implementation facility, the MOVE! coordinator had difficulty assembling the required interdisciplinary team, because service chiefs did not allow staff who willingly volunteered to participate on the MOVE! team:

At the transition facility, we heard about how a key clinical leader succeeded in getting approval for more staff to implement and then expand program:

‘I told them that we wouldn’t play if they didn’t give me the FTE [staff time] […] it came down to, ‘Are we going to do this or not’ and I said, ‘You know, we are more than happy to do this but if you don’t give me the FTE, then you can get dietician involvement by paying somebody from the outside to come in because I won’t do it. I don’t have the staff to support the program’ so I did play a little bit of hardball and put my foot down.’ [Supervisor; 100]

Leadership engagement was often double-coded along with relative priority and available resources. It is sometimes difficult to disentangle these influences. Engagement of leaders is often demonstrated by their actions in reinforcing priority and helping get needed resources in place, as the above illustrations show.

#### Available resources

This construct weakly distinguished between high and low implementation facilities with strong negative ratings in both of the low implementation facilities and a mix of weak ratings in the other facilities. Resources were constrained at all of the study facilities. The most common constraints were lack of dedicated staff time and shortage of physical space. At one of the low sites:

‘There was a lot of conflict with scheduling. The room was only available certain times of the day and it conflicted with other group classes in the room […] we basically moved into a room that was full of storage and we offered to go in there and try to make it conducive to a classroom and once we showed that there was going to be some attendance and it was going to be an ongoing and successful project we were able to get a more permanent location.’ [MOVE!Coord; 500]

At this facility MOVE! was just one more under-funded initiative:

‘Well there’s nothing like an unfunded mandate […] to get […] their blood boiling around here where workloads are so high everywhere else.’ [Clinical Psychologist; 500]

The MOVE! coordinator here purchased supplies out of her own pocket as incentives for Veterans who completed the MOVE! classes until she finally won approval for funding:

‘Our coordinator’s extremely distressed over facility issues and begging […] management has not put money forth for things.’ [Dietitian; 500]

However, staff at the high implementation and transition facilities viewed these constraints as challenges that could be overcome, rather than feeling defeated by them. In fact, one high implementation facility, despite tight budgets, won approval for dedicated staff time and was in the process of hiring an additional staff position that was approved by regional leaders.

#### Access to knowledge and information

This was not a distinguishing construct. NCP published program materials online, including an implementation guide. We coded this construct as ‘missing’ at two facilities because there was no explicit mention of the helpfulness of these materials as an information source and we failed to probe more on this topic. This construct should be distinguished from design quality and packaging, in that it focuses more on access to information about the intervention and how to incorporate it into work tasks; where design quality and packaging focuses more on how components of the intervention itself (such as patient materials) are packaged and presented.

Training is also an important potential source of information and knowledge. No staff at any facility had access to training or training materials to help guide how to implement or administer the program. One high implementation site was rated a weak positive because they had plans to provide training to new staff:

‘Now that I have all the staff hired, we need to set up a face to face training meeting and I myself have never planned a [region-]wide training conference and so they’re helping me get in touch with the education coordinator and helping me with funding to schedule that […] for new MOVE! coordinators and staff.’ [regional Coord; 300]

## Process domain

### Planning

This construct strongly distinguished between high and low facilities, although none of the facilities described a formal planning process for implementing MOVE!. The weak positive ratings for the high implementation and transition facilities were assigned relative to the two low implementation facilities. The former described an incremental approach to implementation and a limited period of collaborative idea generation (*e.g.*, deciding the number of sessions, what content to include in each session) with an inter-disciplinary team. The planning process was disorganized at one facility initially, but a clinical leader set deadlines, which encouraged them to come up with a plan:

‘The Chief of Medicine was in charge of it but didn’t really guide us so much. Just kind of set the deadlines […] it was a little tough, until we finally […] just put it on paper, did it, and started working with it on the fly […] changing things here and there.’ [MOVE!Coord; 300]

The other facility appeared to have a more proactive planning process in place and considered patient needs, ideas from other VISNs, and conference calls with other MOVE! coordinators:

‘Anything new at the beginning was a little overwhelming, trying to set it up, how many weeks we wanted to set up and so forth and we were getting ideas from other VAs and meetings that we were having online and […] teleconferencing within the [region] […] and we [had to] coordinate […] how many weeks we wanted, what topics we were going to address, which people we were going to have.’ [Dietitian; 400]

One low implementation facility recognized the importance of having a plan but struggled to develop one:

‘There has not been to my understanding a really good plan for follow up and that is something I know our coordinator is highly distressed about […] we’ve been weak in having staff time dedicated to be able to do that and so we are saddened by that.’ [Dietitian; 505]

### Engaging

This was not a distinguishing construct. All but one of the study facilities had enthusiastic, skillful, and capable program coordinators committed to getting MOVE! up and running. All facilities were required to name a physician champion; however, these ‘champions’ were largely absent in three of the facilities. The exceptions were physicians at one high and one low implementation facility, who both worked actively to help implement MOVE!

The CFIR explicitly lists several roles within this construct. We did not find any evidence for the role of opinion leaders, and there were no external change agents. It is important to identify other key types of stakeholders prior to conducting interviews, if possible, so that respondents can be asked explicitly about their degree of participation. For example, our study would have benefited by interviewing primary care providers because they are the source of patient referrals to MOVE! but we chose to focus on people more closely involved with implementing MOVE!.

### Executing

As mentioned above, none of our study sites had formal implementation plans, so it was impossible to assess the quality of execution relative to a plan. For example, well-defined incremental milestones were not established, and thus we were unable to assess the extent to which those milestones were met. Thus, we indicate that this construct was non-applicable for all facilities.

This construct is best assessed during the course of implementation, and without formal planning, is difficult to define or measure. It is important to clearly define what constitutes quality execution *a priori*.

### Reflecting and evaluating

This construct strongly distinguished between high and low implementation facilities. One of the high implementation facilities surveyed patients after they completed the program and, based on this feedback, changed the location of the classes. Staff at both high implementation facilities took time to reflect and evaluate in team meetings. To varying degrees, they reflected on how their program could be improved or expanded:

‘Every other Tuesday we do have a meeting with all the members of our group to discuss successes and other things that need to be and this nurse brings us a printout of the patients who were seen and how long they’re seen and what their weight is and which way they’re heading […]—improving or worse in their weight loss and things like that […] we know exactly how many visits the patients had and the success and everything like that […] everyone participates […] we carve out like 20 minutes to 30 minutes maximum to meet, to discuss obstacles, to discuss problems, to discuss you know, things that need to be discussed for us to be able to run this program properly.’ [MOVE!Coord; 400]

## Discussion

### Aim one

To apply the CFIR to identify contextual influences that explain the wide variation in implementation success of MOVE! across VA medical facilities

We evaluated implementation of a weight management program that was disseminated to all VA facilities in 2007. Using a systematic approach based on a qualitative, consensus-based rating process, guided by the CFIR, we found that twelve constructs manifested more positively (ten strongly differentiated, two weakly differentiated) in the high implementation compared to low implementation facilities. Additional file 3 provides recommended actions to improve the influence of each of the differentiating constructs based on our findings.

Only one construct related to program attributes seemed to be an important antecedent to set the stage for successful implementation—staff perceptions of the relative advantage of MOVE! over other alternatives (*e.g.*, status quo, community program). Perceptions about the advantage of MOVE! appeared to be amplified when there was a perception of need for the program (tension for change, a construct from the Inner Setting).

The majority of constructs that distinguished between high and low implementation facilities were related to the inner setting. Klein *et al. *[27] and others [15,28] highlight the important and influential roles and interrelationships of leadership engagement, available resources, and relative priority. Leadership engagement can lead to provision of sufficient available resources in terms of space and dedicated time, and strong communication about the program, which in turn can lead to sufficiently strong perceptions that an intervention has high relative priority in the midst of other initiatives. All three of these constructs had a markedly stronger presence in the high implementation facilities. In addition, MOVE! is a complex program [29] requiring organizational change to deliver a new treatment modality. One indication of the complexity of the program is the need for coordinating efforts of staff across multiple departments [30]. Indeed, we found that high implementation facilities had more high-functioning team, cross-boundary working relationships, and communications about MOVE! (networks and communication). Our findings also affirm the importance of collecting and using available program data as a mechanism (goals and feedback) to engage key organizational leaders and to win approval for staff dedicated time to the program, as well as a process of on-going critique and evaluation (reflecting and evaluating) of the program.

It is important to note unique overarching contextual factors related to implementing MOVE! in VA facilities that may have contributed to the seemingly lack of importance of some constructs. First, there was little variation in the perception of MOVE! itself: four of the five facilities had a positive view of evidence strength and quality, perhaps because NCP, the central coordinating office, assembled and presented results of a pilot study via multiple venues. Adaptability was strongly positive in all but one facility because of the latitude they felt they had to adapt the program to fit their organization, most staff did not think to trial the program before going full-scale, and unless there was a robust pre-existing weight management program already in place, MOVE! was perceived to be a complex program to implement. Second, all facilities had access to program materials, including an implementation guide, power point slides and lesson plans (design quality and packaging) that were developed by an external entity (the National NCP office), which four of the five study sites regarded positively and relied upon to help develop their program. This was likely a significant facilitating factor [31]. Third, a relatively consistent factor that was surely key to any level of success was the enthusiastic, skillful, and capable program coordinators committed to getting MOVE! up and running; all but one facility had such leaders. Fourth, some constructs had a neutral effect across all facilities (*e.g.*, peer pressure, cost) though these constructs may have significant effects in other contexts. For example, none of our study facilities experienced any peer pressure (*e.g.*, competitive market forces) because they are publically funded hospitals. These constructs should continue to be considered in future studies to ascertain their importance in other contexts.

As we continue to use the CFIR in other implementations studies, we plan to build a repository of findings where we can begin to examine the relationships among intervention characteristics, inner and outer settings, and implementation processes to better understand the circumstances under which some constructs have a significant role in affecting implementation, where others do not. Findings from such analyses can then be used for predictive purposes, to help organizations focus on particular constructs over which they have control, given the status of other constructs that are not mutable.

### Aim two

To illustrate how to apply the CFIR to identify influential contextual constructs on implementation and to suggest refinements to the framework, related methods, and directions for future research.

Two of the six team members (LJD and JCL) were familiar with the CFIR, but this study was the first application of the CFIR for guiding data collection, coding, and analysis in a qualitative study. Because we used a consensus-based approach, we did not quantify the number of discrepancies between analysts; but we can report that we had no trouble reaching consensus on final codes and ratings. We did identify a few constructs that were closely related (*e.g.*, relative priority versus patient needs and resources, design quality and packaging vs. access to knowledge and information) and, thus, were more challenging to code; but as a result of our discussions and consensus, we now have specific examples to clarify the distinction among these, which we have added to our Wiki (see below). Our analysts found the CFIR definitions to be sufficiently comprehensive for coding all interview responses. While we did not do a parallel process of inductive coding to compare with our more deductive, framework-based approach using the CFIR, we were open to new themes and the group was comfortable that we did not miss any significant themes outside those captured by the CFIR constructs.

Though ratings were initially assigned within the context of each case, they were also compared across cases. The last step in our analyses used a predominantly variable-oriented approach as the means by which to identify constructs that differentiated between high and low implementation facilities. In essence, constructs were treated like independent variables. A statistical analogy is that these ‘variables’ would have statistical significance in a model predicting implementation effectiveness—*i.e.*, the positive manifestation of any of these factors in a facility improves the likelihood of implementation success. This approach heightens generalizability based on a small sample of cases. However, we are unable to explore interactions between constructs or identify multiple combinations of constructs that may work together for success or failure. Kahwati and colleagues’ evaluation of MOVE! [32] used a qualitative comparative analysis approach which identified combinations of constructs that lead to success or failure. This approach embraces the premise that there are multiple ‘right ways’ that can result in success. A look at the two high and two low implementation facilities in Table 3 shows that each has a different combination of constructs that strongly manifest negatively or positively. We did not have sufficient sample size to generalize from these working combinations.

In our 2009 paper that introduced the CFIR, we posed three questions by which to judge its usefulness as a theoretical framework [16]. The first question is whether terminology and language are coherent. As part of this study we have clarified the operationalization of a number of constructs; and we provide more coding guidance on our online wiki (http://www.wiki.cfirwiki.net). Our analysts, inexperienced in applying the CFIR, were able to successfully use the framework for coding. Second, is whether or not the CFIR promotes comparison of results across studies. Use of a standard set of constructs sets the stage for doing so, but more studies are needed to test this supposition. Last is whether the CFIR stimulates new theoretical development. The CFIR can be regarded as a ‘menu of constructs’ that have the potential to influence implementation [19]. In the case of MOVE! implementation at VA facilities, a working theoretical model to guide future implementation would include the twelve constructs that differentiate between high and low implementation effectiveness. Continued research is needed to develop measures, propose and test models that predict implementation based on these theoretical constructs, and assess the extent to which these constructs can be used to develop implementation strategies to maximize a facility’s chances for success. Some of our recommendations were taken by NCP, the central office administering MOVE!, to bolster implementation in more facilities, but a systematic evaluation of their effect on implementation success has not been conducted. It would also be informative to assess whether or not these same twelve theoretical constructs are predictive in contexts outside the VA or for other clinical interventions.

## Limitations

Our study is limited by several considerations. First, qualitative data was retrospective and elicited from only people directly involved in implementing MOVE!. Other peripherally involved stakeholders (for example, primary care providers who refer patients to the program) were not included. Second, we did not interview patients about their experience but rather relied on impressions from staff. Third, we did not use the CFIR in its complete form; we did not systematically evaluate the individual characteristics domain. We took a decidedly organizational or collective perspective in this evaluation. Thus, our findings do not shed light on how individual characteristics or behaviors may interact with collective actions or perceptions captured by the other four domains of the CFIR. Fourth, though we strongly recommend that analysts be blinded to implementation effectiveness for study during the course of coding and applying ratings to constructs, our analysts were not consistently blinded. Thus, there is the possibility of bias in the ratings. Lastly, our sample size was small (n = 5) and thus generalizability may be limited. However, our small sample size allowed an in-depth analysis and understanding of the implementation experience in five widely varying contexts. Our menu-of-constructs approach, using the CFIR, will promote integration of our findings with findings from other studies using the CFIR.

## Conclusion

This paper presents our approach for using the CFIR to code and rate qualitative data, which can then be used to facilitate comparisons across studies. We are continuing to use the CFIR in this way in our research center’s implementation studies, further refining construct definitions and, in turn, refining and increasing the reliability of the coding and rating process.

An online Wiki resource (http://www.wiki.cfirwiki.net) is available that contains much of the published information about the CFIR and its constructs and sub-constructs. The CFIR Wiki is designed to promote sharing, to elicit suggestions for refinement, and to continue to develop approaches using the CFIR—all of which helps set the stage for synthesis of findings across studies. Guidelines for coding qualitative data are posted along with example interview guides from this and other studies using the CFIR. Registered users can comment on any aspect of the CFIR; *e.g.*, suggesting refinements or changes in construct definitions. We hope that the approach described here and open access to the CFIR will generate on-going dialogue and continued refinement of both the framework and approaches for applying it.

## Competing interests

The authors declare that they have no competing interests.

## Authors’ contributions

LJD and JCL conceived of the paper. LJD drafted the initial manuscript and JCL also wrote significant portions. Both authors read and approved the final manuscript.

## Supplementary Material

Additional file 1 Interview Guide. Click here for file

Additional file 2 Matrix of quotes and memo statements showing manifestion of constructs. Click here for file

Additional file 3 Matrix of potential barriers and recommended actions to overcome. Click here for file
