# Supplementary material for: Evaluation of a large-scale weight management program using the consolidated framework for implementation research (CFIR)
Source: Implement Sci. 2013 May 10;8:51. doi: 10.1186/1748-5908-8-51 (PMC3656778; doi:10.1186/1748-5908-8-51)
Supplement: Additional file 1 — Interview Guide. [file 1748-5908-8-51-S1.doc]

Hello, my name is [name of interviewer]. *Introduce self and other team members in the room.*

- Introduce the project

As you know, the purpose of this Managing Overweight/Obesity for Veterans Everywhere (*MOVE!*) implementation study is to better understand barriers and facilitators for implementing *MOVE!* with the intent to improve implementation of the current program and as pre-implementation work for a future enhanced *MOVE!* implementation study. We want to take this time to talk to you about your experience, so far, with the *MOVE!* program. Your input will help us to evaluate *implementation and variation* in the *MOVE!* program within and between facilities. We want to understand the challenges and successes of adopting and implementing MOVE! so that we can maximize the likelihood that other sites will be successful with this program.

- *Explain the purpose of the interview*

Your interview will help us to better understand the challenges and successes of the *MOVE!* program at your facility. We will be interviewing multiple people at your facility to gain multiple perspectives. We are really interested in learning more about your own experience with this program.

We are going to ask you questions about your experience as the <xxxx> for your facility.

- *Describe the audio recording and how we will assure confidentiality and answer any questions*

This interview will be audio taped so that we have an accurate record of your thoughts. Please be assured that the tapes and your transcript will be kept confidential. Leadership at your facility, any other co-workers, or NCP leadership will not have access to any of your responses nor be able to connect your responses to you personally. Our study does have Ann Arbor IRB approval. Once your interview has been transcribed, only a site identifier will be linked to the transcripts, while any information linking you to the transcript will be destroyed. The audio recording will be destroyed as soon as the transcript is verified and analyzed by research staff.

To help ensure confidentiality, it would help if you would refrain from mentioning the name of your facility wherever possible or mentioning specific names of other staff members during the interview. If, at any time, you feel that the questions are too sensitive, I would be happy to turn off the recorder during that portion of questioning. You may also skip any questions you wish during the interview.

We also wanted to remind you, that we will giving you a $10 Target gift card as a small token of our appreciation for your participation.

Do you have any questions for me? [Answer any questions]

Are you ready to begin? I’m going to start recording now.

*NOTE: Grayed out text are notes to provide extra guidance in using this interview guide. Keep in mind is that this Guide was not used verbatim (except for the closed-ended questions at the beginning)…the purpose of this Guide was to help ensure all topics of interest were covered. We encouraged open narration of participants’ implementation experiences and probed for more details when appropriate. This approach encouraged participants to share information they deemed important, minimized recall bias, and allowed us to more deeply understand their context and implementation process (Belli, 1998).*

**I. INTRODUCTION**

We will turn now to the main part of the interview where our questions will be open ended. We want to hear your thoughts so please do not hesitate to share whatever you believe might be related to any of the topics.

**Now, I would like to ask you a few questions to help me understand your role and who was involved with *MOVE!* at your site.**

**Will you please describe your role within your facility?** Background information

*PROBES*

- What is your title and role within your organization?
- Who do you report to?
- What is your role in the *MOVE!* program?

**Do you network with other clinicians/practitioners who are outside of your facility? If so, will you please describe the nature of those relationships?**

Cosmopolitanism

*PROBES*

- Are you a member of a professional group or society? What level of involvement?
- Do you attend professional or work-related conferences? Outside training?
- How have your network contacts influenced your work?

**What other units/services did you work with to implement *MOVE!*?** Networks & Communications

*Begin with general overview of the “story” of implementation – use structured set of milestones to help guide the story, if possible. This approach will help de-bias recall by using a type of “event history calendar” which will help to promote sequential and parallel as well as top-down retrieval of memories (Belli, 1998)*

**II. OPEN-ENDED DESCRIPTION OF IMPLEMENTATION**

**Now, I would like to hear about the process you went through to implement the *MOVE!* intervention. Later, in the interview, we will talk about particular parts in more detail. For now, though, will you please describe how *MOVE!* was implemented at your site, to the best of your recollection? If you can, please tell me about the major milestones and the month and year in which they each occurred.**

*PROBES*

- Use any background information that might be available in terms of milestone dates, etc.
- How are patients screened for BMI?
- Who determines eligibility? Gives risk education? Offers MOVE!?
- How do patients fill out MOVE!23?
- Who reviews MOVE!23 results with patients?
- Who helps patients set goals?
- Who schedules follow-up MOVE! appointments?
- Who does the telephone follow-up calls? How are calls documented?
- Who tracks patients’ progress?
- How are Level 2 consultations communicated?
- Who leads Level 2 Group Visits? Supporting roles?
- Do you have adequate furniture and equipment? If not, what is lacking?
- Do you have computers for MOVE!23? Printers?
- Do you have adequate exam rooms?
- Do you have appropriate rooms for group visits?
- Do you have a private area for weighing patients?

- Have you established clinic profiles for MOVE! related appointments?
- Are you using clinical reminders?
- Do you have a MOVE! related progress note title in the list of titles

- What kind of marketing activities were done?

|  | Approximate Date Started-Completed | Not at all | | | ½ way | | | Fully Impl. | | | |
| --- | --- | --- | --- | --- | --- | --- | --- | --- | --- | --- | --- |
|  |  | **1** | **2** | **3** | **4** | **5** | **6** | **7** | **8** | **9** | **10** |
| **Screening and determining eligibility** |  |  |  |  |  |  |  |  |  |  |  |
| **Risk education and assessment of readiness to participate** |  |  |  |  |  |  |  |  |  |  |  |
| **Pre-contemplative counseling** |  |  |  |  |  |  |  |  |  |  |  |
| **Level 1: Self-Management Support** |  |  |  |  |  |  |  |  |  |  |  |
| MOVE!23 Questionnaire |  |  |  |  |  |  |  |  |  |  |  |
| Goal-setting |  |  |  |  |  |  |  |  |  |  |  |
| Patient Handouts |  |  |  |  |  |  |  |  |  |  |  |
| Phone/F2F follow-up within 1 week |  |  |  |  |  |  |  |  |  |  |  |
| Phone/F2F every 2-4 weeks thereafter |  |  |  |  |  |  |  |  |  |  |  |
| Connect with resources  Pedometers community |  |  |  |  |  |  |  |  |  |  |  |
| **Level 2: Group Sessions** |  |  |  |  |  |  |  |  |  |  |  |
| Private weigh-in |  |  |  |  |  |  |  |  |  |  |  |
| 1-2 Topical discussions |  |  |  |  |  |  |  |  |  |  |  |
| Group discussions |  |  |  |  |  |  |  |  |  |  |  |
| Individual consultation, if needed |  |  |  |  |  |  |  |  |  |  |  |
| **Level 2: Individual Specialty Consultation** |  |  |  |  |  |  |  |  |  |  |  |
| Referral to dietitian, physical therapist, behavioral health professional, medical specialist, other _________ (as needed) |  |  |  |  |  |  |  |  |  |  |  |
| **Level 3: Weight Loss medications** |  |  |  |  |  |  |  |  |  |  |  |
| Orlistat (Xenical) or sibutramine (Meridia) |  |  |  |  |  |  |  |  |  |  |  |
| Criteria for use |  |  |  |  |  |  |  |  |  |  |  |
| **Level 4: Brief Residential Treatment** |  |  |  |  |  |  |  |  |  |  |  |
| Criteria for use |  |  |  |  |  |  |  |  |  |  |  |
| **Level 5: Bariatric Surgery** |  |  |  |  |  |  |  |  |  |  |  |
| Criteria for use |  |  |  |  |  |  |  |  |  |  |  |
| **Other:** |  |  |  |  |  |  |  |  |  |  |  |
| Multi-disciplinary team approach |  |  |  |  |  |  |  |  |  |  |  |
| Use of maintenance/relapse strategies |  |  |  |  |  |  |  |  |  |  |  |
| Designated facility IT representatives |  |  |  |  |  |  |  |  |  |  |  |
| On-going staff training |  |  |  |  |  |  |  |  |  |  |  |
| Submit annual report |  |  |  |  |  |  |  |  |  |  |  |
| Physician champion |  |  |  |  |  |  |  |  |  |  |  |
| MOVE! Facility Coordinator |  |  |  |  |  |  |  |  |  |  |  |

**Prescribed Implementation Activities**

|  | Approximate Date Started-Completed | Not at all | | | ½ way | | | Fully Impl. | | | |
| --- | --- | --- | --- | --- | --- | --- | --- | --- | --- | --- | --- |
|  |  | **1** | **2** | **3** | **4** | **5** | **6** | **7** | **8** | **9** | **10** |
| **MOVE! Facility Coordinator Appointed** |  |  |  |  |  |  |  |  |  |  |  |
| **Get buy-in from staff** |  |  |  |  |  |  |  |  |  |  |  |
| **Get buy-in from management** |  |  |  |  |  |  |  |  |  |  |  |
| **Measure program outcomes** |  |  |  |  |  |  |  |  |  |  |  |
| **Share information & best practices** |  |  |  |  |  |  |  |  |  |  |  |
|  |  |  |  |  |  |  |  |  |  |  |  |
|  |  |  |  |  |  |  |  |  |  |  |  |
|  |  |  |  |  |  |  |  |  |  |  |  |
|  |  |  |  |  |  |  |  |  |  |  |  |
|  |  |  |  |  |  |  |  |  |  |  |  |

**Some people rely more on formal communication (e.g., email communications through the chain-of-command) while others use informal communication (e.g., a hallway conversation with a co-worker) to accomplish things. Which type of communication has tended to be most helpful for you to accomplish your MOVE! related activities?**

Networks & Communications

*PROBE*

- Focus on informal communications that include unscheduled discussions between staff outside of formal meetings.
- Were the informal communications helpful?
  - If there was not much informal communication, why not? Was the lack of informal communication a detriment?
- Ask for examples related to *MOVE!*.

**III. PRE-IMPLEMENTATION EXPERIENCES**

***INTERVENTION***

**I would like to hear more about how the intervention initially started at your site and how you and others viewed the *MOVE!* program before it was implemented.**

**How did your site become involved with this intervention? How did you personally get involved?**  Intervention Source

*PROBES*

- Would you say the implementation of MOVE! was more externally driven or internally motivated? Why?
- Did someone at your site or you have a say in whether you participated?
- Did you feel like your site’s participation was voluntary?

**What kind of information did you see that showed whether the *MOVE!* program would work?**

Evidence Strength & Quality

*PROBES*

- Information from your own research, consensus guidelines, published literature, or other sources? From co-workers? From supervisors?
- To what degree did the evidence influence your opinion of *MOVE!* before it was implemented?
- Was *MOVE!* a better alternative than other interventions that may have been available at that time?

**To what degree did people involved with *MOVE!*, buy-in to the intervention before it was implemented? Why?**

**Did you see a need for this type of intervention? Why or why not?**

*PROBES*

- At that time, how did you think *MOVE!* would meet that need for clinicians? To what extent did it *actually* meet that need? Relative Advantage…Tension for Change
  - Improvement in work process (amount or type)? Compatibility
- …how did you think *MOVE!* would meet that need for patients? To what extent did it *actually* meet that need?
  - Better patient outcomes? Which outcomes? Patient Needs & Resources

**What kind of services were you already offering to patients who are overweight or obese at your site?** Relative Advantage…Complexity

*PROBES*

- - To what extent were the existing services multi-disciplinary ?
- How were patients being treated?
- To what extent?
- Were there other similar initiatives?
- Do you perceive MOVE as being a superior alternative? Why or why not?
- To what degree was there “competition” for funds, time, or attention because of other initiatives that may have been taking place concurrently?

**IV. IMPLEMENTATION EXPERIENCES**

**Now, I’d like to ask some questions about your experience getting *MOVE!* implemented and the type and level of support you had.**

**On a scale of 0-10, with 0 being very easy and 10 being nearly impossible, how difficult was *MOVE!* to implement in your facility? Why?**- Complexity

*PROBES*

- - What were the barriers you experienced in implementing each of the components?
  - What were the facilitators you experienced in implementing each of the components?

**Can you describe the planning you did to get *MOVE!* implemented?** Process/Planning

*PROBES*

- How did you track what tasks needed to be done? Progress? Status?

**Did you feel like you had enough flexibility (the ability to change aspects of *MOVE!* to make it work) to implement *MOVE!* in a way that would work best at your site? Why or why not?** Adaptability (“Soft periphery”)

*PROBES*

- How could *MOVE!* be more flexible?
- How could *MOVE!* better accommodate unique differences at your site?

**What type and how much IT support did you have when implementing *MOVE!*?**

**Has this sustained over time?** Access to Information & Knowledge

*PROBES*

- Examples (EMR support, *MOVE23!* Patient Questionnaire support)
- Did you encounter technical issues? How were they resolved?

**How is workload reported? Credited?**

- - (Note, the following codes are supposed to be used to report MOVE! workload: 372 Individual Visit; 373 Group Visit; 324+372 or 147+372 telephone. Must be set up as a “count” clinic (not noncount) and must all be “checked-out”

**Will you please describe the physical space configuration used for *MOVE!*?** Available Resources…Complexity

*PROBES*

- What kind of space was used for patient interviews and visits? Group Visits?
- Where were you located (relative to others working on *MOVE!*)?

**VI. OPERATIONAL EXPERIENCES**

**Once *MOVE!* was fully implemented and you started referring patients, how much time and effort was/is required to provide MOVE! on a day-to-day basis?** Complexity

*PROBES*

- Was additional or unforeseen time or effort incurred in getting everything implemented? E.g., IT support, trouble-shooting equipment, etc.
- What resources would be needed to keep *MOVE!* going on a long-term basis?

***EXTERNAL CONTEXT***

**What disciplines or services are involved with MOVE! at your facility?** Networks & Communications

*PROBES*

- Dietitians only? Physicians (which specialties)? Other?
- Describe their involvement.

**Will you please describe the process you used to schedule patients for *MOVE!* visits?** Adaptability

*PROBE*

- What kind of support did you have from IT?
- What kind of support did you have from others? for example, supervisors and support for allocating appointment slots to *MOVE!* patients

**What kind and level of involvement do leaders at your facility have with the *MOVE!* program?** Leadership Engagement

*PROBES*

- What kind of support did they give you? Specific example.
- Did they know about the *MOVE!* study?

**VI. PATIENT EXPERIENCE**

**Why did patients want to participate in the *MOVE!* program?** Patient Needs & Resources … Tension for Change … Compatibility

*PROBES*

- Appeal of the *MOVE!* Program?
- Appeal of 1 on 1 or weekly group counseling sessions?
- What were their goals?

**What did patients think of the *MOVE!* program?** Patient Needs & Resources… Relative Advantage…Compatibility

*PROBES*

- What kind of impact did *MOVE!* have on patients?
  - 1. Opportunity to improve their health?
    2. Motivation to improve their diet or eating habits?
    3. Motivation to exercise more?
    4. Get extra attention?
  - What expectations did patients have in participating in *MOVE!*?
  - How difficult was it for patients to comply with goals?
  - Was age a factor in how patients perceived the program? E.g. under 35?
  - Was gender a factor in how patients perceived the program?
  - Is gender a factor in how patients perceived the program?
  - Is age a factor in how patients perceived the program? E.g. under 35?

**VII. COSTS**

**What kind of funding support did you receive for participating in *MOVE!*?** Available Resources

*PROBES*

- FTE support?
- Money?
- Relief from other duties?
- Were there resources or support that would have helped you implement *MOVE!*, that you didn’t receive?

**Did you have any funding constraints while getting *MOVE!* implemented?** Available Resources

*PROBES*

- Inadequate staffing?
- Inadequate time?
- Competing priorities?

**VIII. OVERALL EXPERIENCE**

**I’d like to switch gears a bit and ask some more general questions.**

**What kind of external support did you receive from your VISN? During Implementation? Since then?** Available Resources

*PROBES*

- Talk about support in terms of mentoring, problem solving, training
- Ask for specific examples.
- Did you have adequate communications with study staff? Externally? Internally?
- Did you feel adequately supported by study staff? Externally? Internally?
- If VISN-level: Are the bi-monthly meetings with NCP helpful? Why or why not?
- *What about MOVE! Program materials distributed via Facility Toolkits and available on the MOVE! Intranet Website:* Examples, the *MOVE!* Clinical Reference Manual, the *MOVE!* Quick Start Manual, the *MOVE!* Pocket guides, *MOVE!* Reference Tools, the Ten Minute *MOVE!* Staff Orientation Video.
- How helpful was this information?

**Generally speaking, what do clinicians at your facility think of the *MOVE!* program?** Individual Knowledge & Beliefs … Evidence Strength & Quality

***Clinicians:* Even without *MOVE!*, do you feel like you are able to get everything done you would like to in a typical day? Why or why not? Is this true for the other clinicians in your unit? Why or why not?** Available Resources…Relative Priority

*PROBE*

- Describe other competing priorities?
- What is the nature of those conflicts?
- Short term? Long term?

***Clinicians only:* How would you describe working relationships between units/services at your facility?**

Networks & Communications

*PROBES*

- How empowered do clinicians feel in doing their work? Interacting with *MOVE!*?
- Do clinicians have close, high-performing relationships?
- How were the relationships between clinicians and IT or other hospital administrative staff or support staff (ie. ADPACs, DSS people, MAS people, clerks, facilities managers (scheduling group rooms, etc.)?
- How do these relationships affect the ability to refer patients, schedule appointments, conduct the interviews, setting things up, etc

**IX. LEVEL OF ENDORSEMENT AND RECOMMENDATIONS**

**On a scale of 0-10, how successful do you think *MOVE!* is at your site? Why?**

Note: We are interested in the “perception” of success; the interviewee can define success in any way.

**If you had the option, would you recommend continuing *MOVE!* at your site? Why or not?**

*PROBES*

- What would it take to keep *MOVE!* going at your site?
- What type of justification would you need to show in order to keep this intervention going over the long-term?

**Would you recommend *MOVE!* to other sites? Why or why not?**

**Do you have any specific suggestions for other sites who have not yet started implementing *MOVE!*?**

**How would you improve the MOVE! Program? Why?**

*PROBES*

- One idea is to develop a web-based interface to provide periodic feedback-perhaps focus on physical activity, using a pedometer to upload step counts so patients can see how they are progressing.

**REFERENCES**

Belli, R. F. (1998). The structure of autobiographical memory and the event history calendar: potential improvements in the quality of retrospective reports in surveys. *Memory, 6*(4), 383-406.
