# Supplementary material for: Evaluation of a large-scale weight management program using the consolidated framework for implementation research (CFIR)
Source: Implement Sci. 2013 May 10;8:51. doi: 10.1186/1748-5908-8-51 (PMC3656778; doi:10.1186/1748-5908-8-51)
Supplement: Additional file 2 — Matrix of quotes and memo statements showing manifestion of constructs. [file 1748-5908-8-51-S2.docx]

**Additional File 1: Manifestation of constructs that differentiated between high and low implementation facilities and insights from the transition facility**

|  |  | **Low** | | **Transition^a^** | | **High** | |
| --- | --- | --- | --- | --- | --- | --- | --- |
| **INTERVENTION CHARACTERISTICS** | | | | |  | | |
| **CIFR Ratings:** | | **-2** | **+1** | **+1** | | **+2** | **+2** |
|  | Relative advantage | One facility preferred a community-based wellness program over MOVE! and referred patients there instead::  *… I have an employee that’s lost almost 100 pounds going through the [Community] Program...their initial statistics are showing almost identical weight loss to the MOVE! pilot study in a 13 week period instead of a six month period. So that was exciting that they were showing some more or even better efficacy because they’re on a shorter time frame than MOVE was offering….if they don’t have a clinic appointment, they do get charged a co-pay… [the community program has] these walking classes, these swim classes, these things are in the community……[and it relies] more heavily on personal responsibility of the participants. They don’t… try to do any oversight or over-bearingness on the participants. The other thing that’s very interesting about the [community] program … is [it] is a wellness program. … the MOVE program has really gone down the road of focusing on obesity.* [MOVE Coord; 200] | | The MOVE! Program emphasized interdisciplinary treatment:  *… we didn’t have all the components that we needed [for weight management]…before MOVE came along, we were in a silo so we did our part as a dietician so we were just working out of our corner of the world…*[MOVE Coord; 100]  MOVE! was viewed as a better alternative than other treatment options:  *…I think they recognize that the magnitude of the effect on the drug therapy is small, in comparison to the surgery but …we used to do those gastric bypasses here but they … stopped them because the morbidity was too high…*[Physician; 100] | | The centrally disseminated MOVE program was an advantage because the top-down “buzz” enabled them to get more disciplines engaged, add to their program, and created visibility for patients to sign up for a more robust program:  *…with the help of MOVE information, MOVE literature, MOVE whatever…it sort of boosted [our existing program] more and we were able to expand more…* [MOVE! Coord; 400]  MOVE!’s emphasis on interdisciplinary treatment was also welcomed:  *…the biggest thing is the inter-disciplinary strength because the dieticians cannot affect the need to change by themselves and even though we’ve been teaching weight control for years and years, the enhanced education for the patients by having those other people up there to support the mental health aspects of this and the exercise aspect as well as our end of it, to me is really the number one* [Supervisor; 300] | |
| **II. OUTER SETTING** | |  | |  | |  | |
|  | **CIFR Ratings:** | **-2** | **0** | **+1** | | **+2** | **+2** |
|  | Patient Needs & Resources | One coordinator believed that patients wanted something more than obesity treatment:  *Anecdotally and speaking very frankly, when I’m at the [Community Program] kickoff which is a very positive high paced environment at the Farmer’s Market…I’m out there with the ‘managing obese veterans everywhere.’ I frankly was a little embarrassed even by the title and had a hard time selling some of our Veterans who came up to the table, who are relatively fit, and trying to sell them that I had something to offer them from a wellness perspective.* {MOVE Coord; 200]  Both facilities mentioned that patients having to pay a co-pay to attend MOVE! classes was a barrier to participating (this co-pay has since been waived):  Surprisingly, some staff thought that patients would be concerned about getting healthier by losing weight and then losing VA benefits as a result:  *…that is a concern for me that there could be this.., “I don’t want to get better because you’re going to take money away from me. I had to fight to get this money and I don’t want to get better” …we’d be shooting ourselves in the foot here.*[Dietitian; 500]  The Coordinator and Champion at one site actively sought and responded to patient requests:  *…we have what’s called the reunion week and we have Veterans who are coming back who started the program over a year ago. They’re able to come in, with the effort I think to motivate those who are in the current group by telling them how they’ve been successful at changing their habits, how they’ve been successful at maintaining their weight loss, how they’ve had improvements in overall health, decreased medications …*[Physician Champion; 500] | | Staff were aware of the inadequacy of their MOVE! program to meet patient needs. They used the fact that patients had to wait 3-4 months to get into MOVE! to make a case for getting dedicated staff:  *…I found out…I have 100 plus consults that are incomplete because I don’t have…help to get them into classes…it’s like 3 to 4 months [wait] and…I showed that we are only meeting about 1% of our population…[and]…I presented three success stories of … I call them my three golden children (laughs) that have lost and done really well in the program to show that it was successful and it wasn’t just a flop program...* [MOVE! Coord; 100]  The backlog causes them to miss the window of opportunity with patients:  *…we have a primary care doctor that gets the patient motivated to lose weight, they put them to the MOVE program and they’re all excited and then they have to wait four months? And by that time, they’re like, ‘Eh, I’m not ready’…*[MOVE! Coord; 100] | | Staff at the high implementation facilities were quite knowledgeable and passionate talking about their Veterans. Staff encouraged participation by family members:  *I have one female veteran right now that she joined the program, she made her husband come…he has come to every class with her….I’ve had grandkids come. I’ve had daughters, sons, certainly a lot of wives and significant others...* [MOVE Coord; 300]  One patient at a high implementation facility was so enthused that he recruited other Veterans to come to MOVE!:  *…and he has this long list of patients. We’re talking about…between 50 and 100 patients…he did it on his own. We didn’t ask him to do this.* [Dietitian; 400]  The MOVE! teams showed patients they cared in many ways and were responsive in designing the program to meet patient needs. One patient was so enthused that he recruited other Veterans to come to MOVE!:  *…and he has this long list of patients. We’re talking about…between 50 and 100 patients and…we asked him not to keep doing that because we can’t get to all these patients… He would take down their name and their last four and he has this long list…he did it on his own. We didn’t ask him to do this.*[Dietitian; 400]  Staff were knowledgeable about characteristics of Veterans including awareness of the discomfort some felt in attending group visits:  *…they don’t quite feel as part of the group if they’re younger than 40. Some veterans that have a BMI between 25 and 30…they feel a little awkward if they’re sitting there at 190 but would like to weigh 170 versus the veteran sitting next to them….Often times the Vets with the BMI younger, under 30 come to a few classes and then they don’t seem to continue…* [MOVE! Coord; 300]  Both MOVE! Coordinators found ways to integrate exercise into the classes based on patient needs:  *It’s one thing to issue a pedometer and say, “Here, walk” but if you get them to actually get in as a group and start walking… I’m going to order stretch bands and provide education to all the MOVE coordinators on how to incorporate exercise into the classes.* [MOVE Coord; 300] | |
|  | **CIFR Ratings:** | **-1** | **-2** | **0** | | **0** | **+1** |
|  | External Policy & Incentives | The absence of performance measures related to MOVE! had a negative affect at both facilities:  *…even more important than [funding] is accountability. Our facility should be ranked/compared/rewarded/admonished based on its relative performance in this program. Anything measured will likely get its due attention/resources, and will likely improve.* [Physician Champion; 500]  In addition, other performance measures seemed to work at cross-purposes to MOVE! because providers did not believe that these measures would be improved by patients participating in MOVE!:  *…there is a performance measure about the lipid profiles and so…that’s what drove the boat…I do not know if there are any performance measures about the weight management issues.* [Former MOVE Coord; 500] | | The absence of a national performance measure seems to work against MOVE! but there was not a clear indication of actual positive or negative influence of performance measures:  *I just think it may require a performance measure…we’re probably going to have a national performance measure on weight management and then at that point, MOVE may really kick in because it’s going to be a critical component of the evaluation of Network Directors and facility directors…* [Regional Coord; 100] | | The MOVE! Coordinator at one facility was working to link MOVE! to changes in physiological measures (e.g., blood pressure), on which existing high priority performance measures relied:  *…I requested…that we would like to have another nurse assigned to our program…to be able to…record information about blood pressures and cholesterols and perhaps blood sugars…I would like to show…that weight loss has something to do with improving all of this.* [MOVE! Coord; 400] | |
| **III. INNER SETTING** | |  | |  | |  | |
|  | **CIFR Ratings:** | **-2** | **-2** | **+1** | | **+2** | **+2** |
|  | Networks & Communications | *Subtheme: Teamness*  There were few to no team meetings or team development despite:  *[if we have any MOVE team meetings] we haven’t been invited. I don’t think we do, though…* [Librarian; 200]  A former MOVE! Coordinator was not told that a new Coordinator had been hired to replace her.  *…the coordinator here of the MOVE! program actually had no idea that we were going to hire someone new. She was not involved even in the process of the fact that my clinical supervisor and my chief of department wanted the other person to do the MOVE! program and that actually did cause some difficulty with the coordinator of the MOVE! program who was not pleased with that transition…she was not involved in the process…she was just sort of told about it….* [Former MOVE! Coord; 500]  *Subtheme: MOVE! Communications*  Some patients did not understand what MOVE! was or why they were referred:  *… sometimes they come without that little hard copy consult and they think that they need to see a movie…they’re kind of confused sometimes about what they’re coming for…I’ve had people say that they were coming because they wanted to lose weight but most…are coming because they were referred to the program.*  *I would have to tell you that he has taken the information to the Patient Health Education Committee about MOVE and I sit on that committee as well so we’re there about the program…*[MOVE Coord; 500]    Some patients were disappointed they were not being referred for bariatric surgery.  *…the Veterans are astounded to hear that we do not do the bariatric surgery here. I get a referral, if I’m lucky I get them because I’m supposed to assess them and then when I start talking about where you’re going to go, most of them tell me, “I didn’t know you didn’t do that here.” I always say, “I can’t believe I’m the one who’s telling you this!”... nobody has…taken them to educate them and sometimes I feel like I’m the only one doing that, thinking there’s something wrong with this.* [Former MOVE! Coord; 500] | | *Subtheme: Teamness*  In the 2^nd^ year, the MOVE! Coordinator assembled an 8-member team who met weekly until MOVE! was running smoothly, phasing down to bi-weekly or monthly meetings.    *Subtheme: MOVE! Communications*  The first year, the Coordinator did not communicate effectively with Service Chiefs about getting their staff involved with MOVE!. The second year, the new Coordinator openly communicated with Service Chiefs about getting staff involved:  *…if we’re going to do it, we do it right. You know, we talked to the service chiefs, we recruit how we need to recruit so we’re on the up and up and we put it in a more positive light…*[Supervisor;100]  The Coordinator had a close relationship with a nurse practitioner who helped her link in with primary care providers:  *I have a nurse practitioner down there and I’m really close and we interact a lot and then she kind of just tells me what the doctors think or what’s going on…they’re so busy, it’s very hard to even meet with them.*[MOVE Coord; 100] | | *Subtheme: Teamness*  Regular team meetings; e.g.,  *…every two weeks we meet after the MOVE sessions…with all the members of our group to discuss successes and other things…we do this through our… supposedly lunch time…we carve out like 20 minutes to 30 minutes maximum…to discuss obstacles, to discuss problems...* [MOVE! Coord; 400]  MOVE! teams coalesced. Multiple members of the interdisciplinary teams confirmed the collaborative nature of their team:  *…from what I’ve seen out in the world…there’s a huge friendliness attitude here…We have a huge staff retention…so we all know each other and have worked together…So that’s a huge benefit for us… nobody was real pushy or bossy or anything. We all kind of collaboratively worked together so that definitely helped.*[Physical Therapist; 300]  *Subtheme: MOVE! Communications*  There was good, cross-departmental relationships:  *If I need something, I just contact either the primary care supervisor or the mental health RN supervisor and request a meeting and they’ve been cooperative.* [MOVE! Coord; 300]  Even the mental health RN at one site was an active member of the MOVE! team:  *I think [relationships are] good [between MOVE team members] because…in the meetings sometimes we find out, maybe that we might be overlapping in what we’re saying…*[MH Nurse; 300]  One Coordinator (a Nurse Practitioner in primary care) had strong working relationships with other primary care providers which helped in coordinating care; e.g.,  *… we had a patient who needed a knee replacement and he was told, “Well you cannot have a knee replacement unless you lose weight”. So of course he lost weight. He lost up to like 60 pounds already but he needs another knee replacement on another leg and he needs to move more…he looks dejected [today], extremely depressed and…he goes, “I’m trying so hard to lose weight and I just saw my primary doctor and my primary doctor put me on diabetic medication and I’m like trying to do all of this and I still have to start taking medication?” so I said, “You know what? Let me go and take a look at your chart…and see what…is actually needed” so I go and review the chart, I call this patient’s primary care provider and I said, “He’s under my supervision and I’m trying to get him to lose weight. Do you agree that we can give him 3 month trial to fix this with diet?” …So he agreed! So I come back to the patient, I said, “Hey, this is what we’re gonna do. You need to meet with dietician, you review your diet, you keep your food diary, we’re going to find out what the deal is and see how we can fix this so that you don’t need treatment, that you can fix it with diet and exercise and weight loss”.*[MOVE! Coord; 400]  Multi-pronged and on-going communications helped to ensure primary care providers continued to refer patients to MOVE!  *… every now and then we’ll send them a blanket message to all providers reminding them about the MOVE! program and how they need to make the referrals to the MOVE! program. I’ve met several times with the LPNs at their monthly meetings, encouraging them as front line people that they need to sell the program and…a lot of them have attended the class to see what it’s like. We have flyers that I’ve put together that are in all the intake rooms where the veterans are waiting for the providers and they can get these MOVE! brochures that explain the program at that point.* [MOVE! Coord; 300] | |
|  | **Implementation Climate** | | |  | |  | |
|  | **CIFR Ratings:** | **0** | **0** | **+2** | | **+1** | **+1** |
|  | Tension for Change | There was no expressed need for the program. | | Staff were frustrated that there was no weight management program to which to refer patients.  … *for a year, it was… stagnant…they had put up the…[MOVE!] posters, …and they didn’t have anything set up so people were consulting to the MOVE program when there wasn’t even a program set up…*[MOVE Coord; 100] | | Facilities stated a general need for MOVE! because of the high prevalence of overweight/ obesity among Veterans. One Coordinator was dissatisfied with their current program:  *…we had nothing else to offer and so they’d attend the class and when you could tell they were newly motivated or wanted additional information, at that point, we had nothing more to offer.* [MOVE Coord; 300] | |
|  | **CFIR Ratings:** | **-1** | **-2** | **-2** | | **+1** | **+2** |
|  | Relative Priority | MOVE! had lower priority at both facilities, relative to other initiatives/issues:  *… we’ve got to pick what’s important to the veteran and to the administration at this point and TBI screening and poly- trauma screening has tended to overshadow the MOVE! program* [MOVE! Coord; 200]  Focus on performance measures meant that MOVE! had lower priority which translated to space not being available for group MOVE! classes because other classes had higher priority:  *… when executive management is being held accountable for certain parameters at the VA…there is a performance measure about the lipid profiles and so …that’s what drove the boat…the big thing, the big conflict…would be space…we have hypertension group classes, we have hyperlipidemia group classes, we have pain management group classes and then there’s MOVE!* [MOVE! Coord; 500]  At the time of our interviews, VA leadership (at the national level) were potting strong pressure on medical centers to reduce clinic backlogs:  *…we had such a backlog…It just depends on where you are on the totem pole….We are absolutely, pardon the expression, under the gun to take care of these returning Iraq veterans and so it’s a matter of, the MOVE program’s important, but these people are on fire over here.* [MOVE Coord; 500] | | The push to reduce backlogs resulted in lower priority for MOVE!:  *…other priorities were more important… at this VA and many other VAs. There’s intense pressure to…see more new patients in the Ambulatory clinics…cut down the waiting time and all that, so…my belief is that that priority trumps the MOVE priority… there’s a shortage of providers, more patients coming in all the time, they’re trying to keep the time to a new patient visit under 30 days… if you go back the last couple of years, there was sort of a slow flow of people coming…out of the military but…I’d say this year, it’s ramped up… if we don’t take care of those guys and girls coming back from Iraq, I mean that is like totally wrong.* [Physician; 100]  The MOVE! Coordinator and her supervisor successfully increased priority for MOVE!, however, by highlighting its backlog:  *… I have 100 plus consults that are incomplete because I don’t have… help to get them into classes. We don’t have any more…employee time dedicated to the MOVE program…I can’t pull any more time from the employees that I’m already pulling … to get into the MOVE program, it’s like 3 to 4 months …I showed that we are only meeting about 1% of our population and… you’re supposed to get a patient to see you…when there’s a consult, within 30 days and we weren’t meeting that.* [MOVE! Coord; 100] | | At one facility, MOVE! (assessments and classes) suffered from lower priority compared to getting a bariatric surgery program up and running:  *Physicians’ attention was on the bariatric surgery program and it was hard to be heard on anything not related to that….We were approved to start a bariatric surgery program, bam, right away and unfortunately all our doctors and administrative people are enormously interested a bariatric surgery so virtually our work in obesity treatment came to a halt and all resources and interests funneled into bariatric surgery, getting that up and running.* [MOVE! Coord; 300]  However, the MOVE! Coordinator successfully linked the success of the bariatric surgery program to increase priority for MOVE! The bariatric surgery program ended up paving the way for MOVE! because it was necessary for bariatric surgery candidate patients to complete MOVE! before qualifying for surgery.  At the other facility, MOVE! seemed to have relatively high priority because PCPs and clinical leaders understood the link between performance measures and losing weight. They believed having a viable weight management program was an important component of clinical care:  *…when they first started, they said we’re going to start the MOVE program and I was really skeptical and said, ‘Okay let’s see how long this lasts here’ but then they said it was going to be a nationwide effort…and then once we started actually meeting with other staff, doctors, and, nurses, everybody’s involved so we had this interdisciplinary approach and in my mind I said, ‘Okay this is something that, this is going to kick off and we have to be serious about this. This is something we’ll have to start working at and trying to implement it.* [MOVE! Coord; 400] | |
|  | **CIFR Ratings:** | **-2** | **-1** | **+1** | | **+1** | **+2** |
|  | Goals and Feedback | One facility referred patients out to a community-based wellness program but did not track participation or outcomes:  *…when you enroll people and you know, you can’t be calling everybody and saying, “Are you accomplishing your goals” I mean…it would be nice if you could.* [MOVE Coord; 200]  There was no infrastructure for data reporting. The Coordinator was tracking aggregate data but managers seemed uninterested until recently, the Coordinator’s supervisor, asked for data to begin building a case to expand the program:  *…the group that just completed, lost 96 pounds in 10 weeks and that was a total of probably 9 people I think… We keep a log of all the patients and we also do a fat percentage. We also do girth measurement and we tell them at the beginning, we do a BMI calculation at the beginning and at the end…my direct supervisor has asked for it… and hopefully we can get another class…. I haven’t pushed it because our clinical responsibilities are so high and pulling it together, we’ve worked on it every spare minute for the past two days…just compiling the data on a hard copy...* [MOVE! Coord; 500] | | The team is motivated by anecdotal results but has not implemented regular program tracking.  *I just tracked them from the beginning to what the pre, or what their current weight was…* [MOVE Coord; 100]  *…it’s really been successful from what I’ve watched with the veterans… just people getting an understanding of what they’re going to need to do to have continued success to maintain weight reduction and lifestyle change and just the individuals as a whole, being able to connect with each other and it really builds a support base and just in terms of data that our coordinator had shared with us, some of the individuals are really making strides with their weight reduction.* [Behavioral Health; 100]  Data and anecdotal stories were used to win approval for dedicated staff by showing alignment with the organizational goal of reducing clinic backlog as described under “Relative Priority” | | Both facilities tracked program data regularly and multiple stakeholders were aware of program performance as a result:  *I know how they’re doing during my MOVE Level 2 classes because I keep track of their weight from week to week…We do every quarter look at all the surveys and my clerk kind of comes up with a report of all the questions, comments, outcomes and I send that to [the Physician Champion] quarterly.*[MOVE Coord; 300]  Data are tracked manually by the Coordinator or her supervisor:  *When we have our meetings…we get statistics on that…who gained, who lost, who stayed the same and so forth…*[Dietitian; 400]  The regional-level coordinator coached a facility-level coordinator on how to use numbers to make a case for getting more resources for the program:  *I feel so bad for her because her numbers look so bad …she takes it very personally…and I’m like, ‘Use these bad numbers to your advantage.’ Go to people and say ‘Look, I’m one little person, look what I’m doing. Look what they’re doing down the block’ …and she didn’t realize that until I put it that way. She’s like, ‘Oh I never thought of it that way’. I’m like, ‘This is not a poor reflection on YOU, this is a poor reflection on the fact that the people who you work with aren’t supporting you in the program.’* [Regional Coord; 400] | |
|  | **CIFR Ratings:** | **Missing** | **-1** | **Missing** | | **+1** | **+2** |
|  | Learning Climate | An important dimension of Learning Climate is feeling psychologically safe to take risks for the sake of getting a program up and running. There were indications at one facility that may not have been present:  *… I contacted the next likely person… he just seemed to be so enthused about our goals…[so I] Focused him in my binoculars…I sent him an email. I didn’t want any arrows in my back so …the safest thing to do here is in the little email (laughing) and then if that gets positive response, then you actually meet someone.* [MOVE! Coord; 500] | | We heard several stories that indicate taking risks and innovating are not encouraged in this relatively bureaucratic culture though there were no direct quotes. | | Both facilities exhibited features of a learning climate: 1) MOVE! Coordinators were not afraid to experiment; 2) they shared ideas with peers and superiors; 3) they had regular forums through which to learn from others. For example, one Regional Coordinator rotated meetings between sites in the regions so people could get to know one another and their contexts. Facility Coordinators kept in touch with one another through email and phone as well:  *…[these connections] give me an idea of what they’re doing and how we can modify here and I can give them a few suggestions that we have…we kind of share, exchange information and it really benefits both sides because people do things differently and we learn from each other.* [MOVE Coord; 400] | |
|  | **Readiness for Implementation** | | |  | |  | |
|  | **CIFR Ratings:** | **-2** | **-1** | **+2** | | **+2** | **+2** |
|  | Leadership Engagement | Leaders at one facility were actively against implementing MOVE! and did not see it as a priority even while developing a bariatric surgery program (which requires surgery candidates to go have completed a less-intensive self-management support program such as those provided through the MOVE! program components provided at our five study facilities).  At the other facility, the MOVE! Coordinator had difficulty building an interdisciplinary team because service chiefs were not supportive:  *…when the MOVE came along, there were other people who volunteered to do MOVE. Their supervisory staff did not agree that that was a good idea so then there was the question of who would do it so it turned out that no one actually could set aside their time to do it so that’s why I adopted it because we needed to start a MOVE program and we were late in the game starting it.* [MOVE Coord; 500]  Two different physicians were unable to take on roles related to MOVE!; the implication being that their superiors did not support their involvement with MOVE!:  *We’d had another physician who showed a lot of interest and had volunteered to do that but he was more supervisory staff… and due to time constraints and his schedule, he, after about two months of looking over the literature, he gave it all back to me and said he didn’t think he could do it… then shortly after that, our provider who said she would be the MOVE coordinator came to me and said she couldn’t do that…*[MOVE Coord; 500]  Leaders were uninvolved even after MOVE! was up and running:  *…nobody is directly communicating with me from leadership so as far as I know, they pretty much let us go and do our own thing.* [Physician Champion; 500]…though his supervisor did allow 3 hours/week for MOVE!. | | The Medical Center Director seemed to take a check-the-box approach to MOVE! at first with little engagement. However, the Director was congratulatory about a related project which helped increase visibility of MOVE!.  MOVE! Coordinator’s supervisor was especially engaged and supportive in active ways:  *… I told them that we wouldn’t play if they didn’t give me the FTE [staff time] …it came down to, “Are we going to do this or not” and I said, “You know, we are more than happy to do this but if you don’t give me the FTE, then you can get dietician involvement by paying somebody from the outside to come in because I won’t do it. I don’t have the staff to support the program” so I did play a little bit of hardball and put my foot down… we went through a restructure and at that point, I went to the Resource Management Board and put together a full time FTE request and .5 of that was a GS-11 MOVE coordinator/dietician so that’s how the job was kind of posted. And that was… the only way I was going to go forward with MOVE and my support so I got a .5 and brought our current coordinator on board … it’s been so effective and well received…that I went back to Resource Management Board…requesting a full time GS-12 MOVE coordinator…so I could expand the program to the other two segments of outpatient population plus specialty clinics plus eventually hitting all 7 CBOCs [Community-based Outpatient Clinics]. And that’s why I said leadership has bought in because they just don’t approve positions.*[Supervisor; 100]  This heightened priority helped get more staff time for the program. For example,  *I met with the chief of the mental health department in our facility a couple times and he knew it was a priority but they didn’t have any… extra staff that he could give me and so I just kept at him and finally I told my chief … “I have to go to the [Medical Center] Director because we have to get this started and I can’t start it without a behavioral health counselor”…I didn’t even have to go to the Director, I guess I just had to say it and then all of a sudden, I had a phone call and said, “Okay we’ve decided that we can give you this person”…* [MOVE! Coord; 100] | | Leadership and lower level managers/ supervisors were engaged and committed to making MOVE work. They supported getting people on the interdisciplinary team. The Coordinators had supervisors who were actively supporting the program/implementation.  *I believe at that point back in ’05 I was a .8 FTEE so I had every Friday off [I: Okay] and my supervisor just came to me and asked if I’d be willing to go full time and tackle this MOVE coordinator position as a .2… and of course it immediately was way more than 20%... Slowly I was able to cut back on some other classroom teaching duties [and my supervisor was] always very supportive* [MOVE Coord; 300]  *Our Chief of Medicine was the, I guess they call her what, the champion or whatever of the MOVE program and she did get at the end, kind of like pushy a little bit like, “Let’s get going” …when we were given the two MOVE positions in April, the chief of medicine opted to go with a MOVE RN and the MOVE coordinator… and the Chief of Medicine was in charge of it but didn’t really guide us so much. Just kind of set the deadlines…*[MOVE Coord; 300]  *…if we have any equipment issues or you know, space issues, although space, you know, is hard but you know, they continue to look for us and help and it’s kept up there on the, I would call the radar, so they haven’t forgot about it. They’re, you know, if you say, “Can you bring it up at this meeting” and that, they certainly will…Yep and our prevention coordinator asked about the MOVE program on the conference calls which are VISN calls so I would say leadership here is supportive and interested in it and then by them agreeing to hire, to hire a two positions for this MOVE program I think says a lot. It’s saying yes we will support you, we have value into the program.* [Supervisor; 300]  Both VISN Coordinators were also actively involved. One kept the MOVE program visible at the VISN level.  *…a lot of [getting approval for 9 FTES] has to do with how you present your outcomes and your successes. You just have to keep touting that you’re doing a good thing and sell the confidence that you can continue to do your job if, and you’re going to increase the program.* [Regional Coord; 400] | |
|  | **CIFR Ratings:** | **-2** | **-2** | **-1** | | **+1** | **-1** |
|  | Available Resources | Additional resources (e.g., staff time, funding, space) were not provided to facilities to implement MOVE!. Thus, resources were constrained at all of the facilities in our study. | | | | | |
|  |  | Constraints in monetary resources were mentioned at both facilities. For example,  *Well there’s nothing like an unfunded mandate...to get...their blood boiling around here where workloads are so high everywhere else.* [Clinical Psychologist; 500]  At the other facility, until the Hospital Director finally approved funds, the MOVE! Coordinator purchased supplies out of her own pocket as incentives for Veterans who completed the MOVE! classes:  *Our coordinator’s extremely distressed over facility issues and begging…management has not put money forth for things.* [Dietitian; 500]  Dedicated staff time was also a frequently mentioned resource constraint along with lack of space for holding the group classes:  *We had wanted to start earlier in the spring [sic]. There was a lot of conflict with scheduling. The room was only available certain times of the day and it conflicted with other group classes in the room…we basically moved into a room that was full of storage and we offered to go in there and try to make it conducive to a classroom and once we showed that there was going to be some attendance and it was going to be an ongoing and successful project we were able to get a more permanent location.* [MOVE! Coordinator; 500] | | In early attempts to implement MOVE!, resources (especially dedicated time from staff) were severely limited and despite recent success in getting approval for additional dedicated staff time, they were not able to get physician time dedicated to the program and they lack funds for patient tools. Because of program size constraints, they were able to take referrals from only one of their primary care clinics at the time of the interviews but planned to expand with the help of staff newly approved for the program. | | The climate at both facilities was that though resources were constrained, the MOVE! Coordinators viewed these constraints as something that could be overcome, rather than being defeated by them.  One facility was unusual in having explicitly approved and dedicated staff time up front, with even more staff time recently approved at the region level. However, they were still struggling because they had been waiting months for positions to be backfilled so new staff could work with MOVE!.  At the other facility, the MOVE! Coordinator had to make a case over time for dedicating increasing amounts of her time to MOVE!. She also succeeded in negotiating dedicated time for other staff with their respective Service Chiefs to assemble the multi-disciplinary team. | |
|  | **CIFR Ratings**: | **-1** | **Missing** | **+1** | | **+1** | **+1** |
|  | Planning | Planning at one site was trial and error, not a well thought out plan. Any planning activities were focused on getting a bariatric surgery program up and running. When they hire more people, and coordinate better with MOVE, they “can move forward with more robust programming”:  *…we’re actually advertising for a bariatrics coordinator position which will be a dietary nutrition position and that person would also become the MOVE coordinator and that would be part of their FTEE and at that point, hopefully we can move forward with more robust programming.* [MOVE! Coord; 200]  The other site relied on efforts of the coordinator working without a formal plan. She was also unable to plan for follow-up care because of lack of staff:  *…there has not been to my understanding a really good plan for follow up and that is something I know our coordinator is highly distressed about… we’ve been weak in having staff time dedicated to be able to do that and so we are saddened by that.* [MOVE! Coord; 500] | | The new MOVE! Coordinator described a general plan for implementation though specifics were not available:  *Before I came to [Study Site] from [Another Site] VA, they had tried to start the MOVE program probably like a year before I got here and I felt like in that time, that they just wanted to get it started and they didn’t really have what they were going to do about it and then I got here and I took it over and then from that point on, because I had some kind of previous experience with MOVE! that once I got here and got everything in order, we started it and it was better.* [MOVE! Coord; 100] | | Neither facility had formal plans in place.  The planning process was disorganized at one facility initially but the COM did set deadlines and then they came up with a plan:  *…the Chief of Medicine was in charge of it but didn’t really guide us so much. Just kind of set the deadlines…it was a little tough, until we finally…just put it on paper, did it, and started working with it on the fly…changing things here and there.* [MOVE Coord; 300]  One team member at one facility reflected that it would have helped to have more time to train or orient themselves before launching into implementation though NCP’s materials were quite helpful:  *… it would’ve been nice to have time to sit down and do more, read more materials, get that [up front development time]…it was helpful to have the worksheets …they do have a bunch of handouts and those were given to us but then just getting the time to look over that and develop it before you stepped in.* [Mental Health RN; 300]  Their Regional Coordinator discussed plans for a future training conference:  *…now that I have all the staff hired, we need to set up a face to face training meeting and I myself have never planned a VISN wide training conference and so they’re helping me get in touch with the education coordinator and helping me with funding to schedule that…for new MOVE coordinators and staff.* [Regional Coord; 300]  The other facility appeared to have a more proactive planning process in place and considered patients needs, ideas from other VISNs, and conference calls with other MOVE! Coordinators:  *… anything new at the beginning was a little overwhelming, trying to set it up, how many weeks we wanted to set up and so forth and we were getting ideas from other VAs and meetings that we were having online and…teleconferencing within the [region]…and we [had to] coordinate …how many weeks we wanted, what topics we were going to address, which people we were going to have…*[Dietitian; 400] | |
|  | **CFIR Ratings:** | **-2** | **+1** | **-1** | | **+2** | **+2** |
|  | Executing | One facility did not implement MOVE!, nor were they following up with patients:  *If you follow the MOVE prescription, somebody’s supposed to be calling that veteran back at 1, 3, and 6 weeks I think and saying, “How are you doing on those goals that we agreed upon?” or whatever. I don’t think that’s getting done and I don’t think it’s getting done at our facility or anywhere else in our network.* [MOVE! Coord; 200]  The other facility struggled with implementation but did implement many (though not all) components of the program. | | This facility took an incremental approach to implementation with MOVE! only available to a single primary care “firm:”  *…and so that was another point in my proposal, you know, we’re not even reaching you know, hardly any patients because we’re only really looking at the Firm A patients…we’re also looking at having more than two orientation classes once we have more FTEEs to go toward that…and then we could open it up to the other firms… I didn’t want to open it to everyone and have like 1000 consults waiting for me… so that’s what we did here just so that it wouldn’t be overwhelming for not just me but for the patients too because…a six month wait time would be awful.* [MOVE! Coord; 100] | | Both facilities had robust and relatively comprehensive MOVE! programming and both took an incremental approach to implementing the full program:  *For approximately a year, we had Level 1 and Level 2 only and then a year ago, …we opened our Level 3 clinical time running.* [Physician; 400] | |
|  | **CFIR Ratings:** | **-1** | **-2** | **+1** | | **+1** | **+2** |
|  | Reflecting & Evaluating | Neither facility had time or space in which to reflect on success or issues related to MOVE! nor were the programs actively evaluated. However, monthly regional phone calls with other facility and the regional coordinators does provide limited time and opportunity for reflection:  *…nobody is directly communicating with me from leadership so as far as I know, they pretty much let us go and do our own thing. On a national level, I know that they are asking for feedback and numbers and reports…I am unaware of anyone on a local level asking for similar information about the program.* [MOVE! Coord; 500] | | There was indirect indication of reflecting on the program that resulted in improvements:  I think they’re doing a better job now of actually talking to the individuals about MOVE before they just enroll them in it. Because I think at the beginning, especially some of the groups were kind of rough because people just got referred and you know, some of these individuals were just referred by their you know, primary care doctor and they really weren’t that, you know, overweight at all and they just happened to be referred because their BMI seemed to be higher but some of them, you know, were muscular people who were heavier… and then also really checking, what are peoples’ motivations for MOVE and I think now they’re doing a better job of that but in the beginning, that was an obstacle.  But they are meeting regularly about the program:  *The three of us meet probably, at the beginning it was like twice a month but now we probably meet once every six weeks…and…since we got this proposal, we’re going to try to add more in …all the time we meet and we try to figure out how we can better the program.* [MOVE! Coord; 100] | | Staff at both facilities take time to reflect and evaluate in team meetings. To varying degrees, they knew the data and reflected on how program could be improved/expanded:  *…on Tuesday after our sessions, every other Tuesday we do have a meeting with all the members of our group to discuss successes and other things that need to be and this nurse brings us a printout of the patients who were seen and how long they’re seen and what their weight is and which way they’re heading ...— improving or worse in their weight loss and things like that…we know exactly how many visits the patients had and the success and everything like that… everyone participates…*  *we carve out like 20 minutes to 30 minutes maximum to meet, to discuss obstacles, to discuss problems, to discuss you know, things that need to be discussed for us to be able to run this program properly.* [MOVE Coord; 400]  Both facilities actively elicit feedback from patients and make changes based on that feedback.  One facility had active and on-going participation of a clinical leader in assessing and improving the program but this was not happening at the other facility:  *…[Chief of Medicine and I] have very little contact…We do every quarter look at all the surveys and my clerk kind of comes up with a report of all the questions, comments, outcomes and I send that to her quarterly…I did meet with her and one of her assistants just a couple of times just to basically discuss what we wanted the MOVE RN to do and I really have had very little contact with her all summer since we did that.* [MOVE Coord; 300]  One of the high implementation facilities surveyed patients after they completed the program and based on this feedback, changed the location of the classes:  *At one time, we had [the class] in the auditorium and…we are constantly trying to get feedback from [patients] and how they feel with it and that was too big so that’s why we went to two groups instead of the one…I think the patients, requesting are seeing [our] interest in them…*[Mental Health RN; 300] | |

1. The transition facility failed in their first year’s attempt to implement MOVE!. After that failed attempt, key changes were made and by the time of the interviews, this facility was well on their way to successful implementation of MOVE!. Information included under this column highlights insights gained through this “before-after” glimpse into their implementation process.
