# Supplementary material for: Evaluation of a large-scale weight management program using the consolidated framework for implementation research (CFIR)
Source: Implement Sci. 2013 May 10;8:51. doi: 10.1186/1748-5908-8-51 (PMC3656778; doi:10.1186/1748-5908-8-51)
Supplement: Additional file 3 — Matrix of potential barriers and recommended actions to overcome. [file 1748-5908-8-51-S3.docx]

**Additional File 3: Potential Barriers and Recommendations to Overcome Them**

|  |  | **Potential Barriers** | **Recommended Actions**  **(Best Practices)** | |  |
| --- | --- | --- | --- | --- | --- |
| **INTERVENTION CHARACTERISTICS** | | | |  |  |
|  | Relative advantage | - A community-based program which focused on wellness rather than obesity, was regarded as a superior option. | - Facilities had the latitude to utilize other program approaches as long as they offered weight management support but when doing so, it is important to track program data (e.g., weight loss) for Veterans participating in those programs. - NCP (a systems-level entity) collects and disseminate data related to MOVE!, highlighting best practices, success stories, and overall effectiveness. Locally, use intentional communication strategies to get this and local data into the hands of clinical leaders. | |  |
| **II. OUTER SETTING** | |  |  | |  |
|  | Patient Needs & Resources | - MOVE! may be perceived as a program for obese Veterans which may cause Veterans to feel stigmatized by participating. - Out-of-pocket co-pays from patients may be a barrier. - Some staff and patients may be concerned about patients losing benefits if their health improves after losing weight. - Patients may need to wait for several months to start the program because of large backlogs. | - Emphasize MOVE! as a wellness program focused on maintaining healthy weight. - Welcome “significant others” (spouses, etc) into the program. - Backlogs can be shortened by allowing participants to join MOVE! at any point in the program. Veterans are more likely to participate if they are able to start the program shortly after their primary care provider refers them. - Actively elicit input from Veterans about how the program can be improved and be clear that implemented ideas came from participants. - Consider forming groups for Veterans who may be uncomfortable participating in a group or who may have unique challenges or concerns; e.g., women, OEF/OIF (younger) Veterans. - Get successful patients active in the program e..g, orienting other Veterans to the program, sharing their success story - Incorporate physical activity (e.g., walking) into the program. | |  |
|  | External Policy & Incentives | - Lack of performance measures for MOVE! may relegate the program to a lower priority than other initiatives that are linked to a performance measure. | - Collect data about MOVE! participants to track improvements in e.g., blood pressure, LDL cholesterol that are often high-priority performance measures. - In VA, national performance measures are an integral component of executive leadership evaluations. VA has implemented the following performance measures for MOVE!:   - Screening for overweight/obesity and offering MOVE!   - Participation in MOVE! if screened positive   - Intense and sustained treatment (at least 8 visits over a 4 month period) | |  |
| **III. INNER SETTING** | |  |  | |  |
|  | Networks & Communications | - Lack of interdisciplinary team formation. - No time for team meetings. - Patients may be confused about what MOVE! is - It may be challenging to get primary care providers to continue to screen for obesity and refer candidate patients to MOVE! - Lack of communication across professional and/or service line boundaries | - Develop a plan and be clear, up front, about how much time is needed and negotiate dedicated time needed for the program with supervisors. - Set up regular meetings – more frequently during initial implementation – to provide face-to-face time for collaborative program development, problem-solving, and “teamness” - Acknowledge that you are a team, take time to build quality relationships with everyone on the team, and work to get members committed to completing team tasks and achieving goals. Many team-building resources are available. One resource available to VA staff, is the Team Development Measure which can be used to assess your team’s performance and develop strategies to improve: <http://www.queri.research.va.gov/ciprs/projects/ResourceGuideV1-1.cfm> (look toward the bottom for the “Module: Team Development Measure” for the guide. Other resources are available here as well. Another comprehensive resource is available through the Agency for Healthcare Research and Quality (AHRQ): <http://teamstepps.ahrq.gov/>. - Define clear roles and responsibilities. - Keep Service Line Chiefs and other key leaders informed of staff involvement with MOVE! and of program successes - Use multiple communication channels (regularly and sustained over time) to help ensure providers and patients understand the program; e.g., flyers and/or brochures in high-traffic areas, agendas for staff and leadership meetings, meet with front-line nurses/LPNs regularly. - Most MOVE! programs were administered through Food & Nutrition Services. However, programs administered by primary care are better positioned to co-manage patients with obesity-related conditions e.g., adjusting medications for hypertension, blood glucose for those who successfully lose weight or increase physical activity. | |  |
|  | Tension for Change | - Stakeholders may not perceive the need for MOVE! | - Use administrative data to show the high prevalence of overweight/ obesity among local Veteran population. This is also confirmed in the literature. Highlight the fact that many Veterans are continuing to gain weight over time, including newly returned Veterans, e.g., OEF/OIF. - Highlight aspects of MOVE! that may address gaps in existing programs. | |  |
|  | Relative Priority | - MOVE! may have lower priority compared to other initiatives happening at the same time - Clinicians may not believe lifestyle change will impact high priority performance goals such as controlled blood pressure. | - Link MOVE! to other high priority initiatives, e.g., requiring participation in MOVE! to qualify for more intensive weight management (e.g., bariatric surgery) or highlighting long backlogs and the need to expand/support the program to reduce the backlog. - Show how change in weight or physical activity change, e.g., blood pressure, lipid measures using evidence from the literature and if possible, augmented with data from patients who complete your own program. | |  |
|  | Goals and Feedback | - It is challenging to track patients’ weight and other measures over time. Often staff lack time and ability to analyze these data and develop cogent business cases needed to support the program. - It is especially challenging to track patients who are referred to a community-based program. - Lack of knowledge of organizational goals and alignment of MOVE! with those goals. | - Local facility staff can access aggregate and patient-level data, e.g., visits, weights for their program using centrally or regionally developed data management tools and reports, e.g., through regional data warehouses. Actively track these data to monitor where improvements may be needed, identifying program gaps, and/or making a business case for expanding the program if/when needed. Link data to high priority organizational goals as described under Relative Priority. - Collect and share anecdotal success stories that help to bring data “alive” for leaders and other stakeholders. | |  |
|  | Learning Climate | - Some organizations do not provide a psychologically safe environment for taking the risks that may be necessary to get a complex program implemented. | - Identify a leader who can help break down barriers and “protect” staff from possible repercussions of failed experiments and who is willing to listen and provide feedback on plans and ideas. - Regional Coordinators can help build a positive learning climate by developing quality working relationships between Coordinators at facilities in the region. Successful strategies include rotating face-to-face meetings between facilities when possible and holding regular conference calls that actively encourage participation from everyone. | |  |
|  | Leadership Engagement | - Clinical leaders and/or executive leaders may not support weight management as a high priority clinical program. - Service line chiefs may have conflicting priorities and not be willing to dedicate staff time to the multi-disciplinary MOVE! team. - Leaders may not provide material support for the program. | - Clearly and explicitly dedicate staff time to MOVE! - Regional and local leaders keep MOVE! visible by regularly including in staff agendas: highlight success stories, track progress - Actively communicate program data and success stories - Clinical leaders can encourage providers (in staff meetings and informally) to refer patients to MOVE! and explain why they are being referred. This should be done regularly – one time is not enough. - Coordinator and team members communicate with their supervisors and other leaders on the topics above (manage upward). - Coordinator and team members keep service line chiefs apprised of program needs and successes. | |  |
|  | Available Resources | - Severe constraints on space in which to conduct group visits are common - Severe constraints in dedicated time to administer the program are common | - Heightening Relative Priority, Tension for Change, and Leadership Engagement may be the most promising strategies to gain commitment of time and space needed to support MOVE! - Build a business case for the program to get the dedicated time and space needed. | |  |
|  | **V. Process** | |  | |  |
|  | Planning | - None of the facilities had a formal, comprehensive plan in place. | - Conduct assessment of potential barriers to implementation - Visit or talk on the phone, with Coordinators and leaders at other successful sites - Develop a plan for implementation with clearly stated aims, timeline, roles and responsibilities, and goals.   - Tailor implementation strategy to identified potential barriers   - Leverage facilitating influences e.g., involve a committed leader early in the planning process.   - Include comprehensive engagement strategy for stakeholders including providers, clinical leaders, service line chiefs, potential team members, etc | |  |
|  | Executing | - Few facilities took an incremental approach or trialed the program. | - Implement MOVE! incrementally and communicate early successes | |  |
|  | Reflecting & Evaluating | - MOVE! team members have inadequate time and supporting tools or infrastructure to evaluate or reflect on progress of the program. | - Arrange for regular team meetings to review progress in terms of process (e.g., enrollment) and outcomes (e.g., weight loss) - Budget time to purposively reflect on and review progress and brainstorm improvements. | |  |
